# Systems view of adipogenesis via novel omics-driven and tissue-specific activity scoring of network functional modules

**DOI:** 10.1038/srep28851

**Published:** 2016-07-07

**Authors:** Isar Nassiri, Rosario Lombardo, Mario Lauria, Melissa J. Morine, Petros Moyseos, Vijayalakshmi Varma, Greg T. Nolen, Bridgett Knox, Daniel Sloper, Jim Kaput, Corrado Priami

**Affiliations:** 1 The Microsoft Research – University of Trento Centre for Computational and Systems Biology, Piazza Manifattura 1, 38068 Rovereto, Italy; 2 Division of Systems Biology, National Center for Toxicological Research, FDA, 3900 NCTR Road, Jefferson, AR 72079, USA; 3 Nestlé Institute of Health Science, Lausanne, Switzerland; 4 Department of Mathematics, University of Trento, Via Sommarive 14, 38050 Povo, Italy

## Abstract

The investigation of the complex processes involved in cellular differentiation must be based on unbiased, high throughput data processing methods to identify relevant biological pathways. A number of bioinformatics tools are available that can generate lists of pathways ranked by statistical significance (i.e. by p-value), while ideally it would be desirable to functionally score the pathways relative to each other or to other interacting parts of the system or process. We describe a new computational method (Network Activity Score Finder - NASFinder) to identify tissue-specific, omics-determined sub-networks and the connections with their upstream regulator receptors to obtain a systems view of the differentiation of human adipocytes. Adipogenesis of human SBGS pre-adipocyte cells *in vitro* was monitored with a transcriptomic data set comprising six time points (0, 6, 48, 96, 192, 384 hours). To elucidate the mechanisms of adipogenesis, NASFinder was used to perform *time-point analysis* by comparing each time point against the control (0 h) and *time-lapse analysis* by comparing each time point with the previous one. NASFinder identified the coordinated activity of seemingly unrelated processes between each comparison, providing the first systems view of adipogenesis in culture. NASFinder has been implemented into a web-based, freely available resource associated with novel, easy to read visualization of omics data sets and network modules.

High-throughput technologies have enabled biologists to produce unprecedented quantities of data ranging from gene expression to protein abundances and metabolic profiles. The tables of numbers and annotations produced by this screening of biological systems need to be interpreted in order to turn data into actionable knowledge^1^. Network analysis is an increasingly prevalent tool for representing the complexity of biological processes and how they relate to each other^2^,^3^,^4^,^5^. Identification of active sub-networks (modules) based on omics data sets can assist in explaining properties of the underlying biological system^6^. Networks are also used to integrate multi-source and multi-level data sets to improve the quality of the biological interpretations^7^. The quality of the results, and therefore functional interpretation of high dimensional data sets, must account for differences in biological processes and functions within different tissue and cell types, which is now possible with available databases of protein and RNA abundances in over 40 tissues^8^.

Adipocyte differentiation or adipogenesis is the process by which pre-adipocytes differentiate into mature functional adipocytes by accumulation of triglycerides^9^,^10^,^11^. This process involves a complex sequence of events that occur in a coordinated fashion for driving the differentiation of preadipocytes with a fibroblastic morphology to fully differentiated rotund fat-storing cells. Adipogenesis is a protective mechanism whereby the excess energy and calories consumed gets stored as fatty acid in adipocytes. The storage capacity of adipocytes prevents ectopic accumulation of fat in other organs such as the liver, skeletal muscle, heart, pancreas, or other tissues^12^ that can result in the development of insulin resistance. Unconstrained adipogenesis can result in the expansion of adipose tissue and the development of obesity^13^. A number of different factors can trigger adipogenesis resulting in increased adiposity including nutritional overload^14^, environmental contaminants and xenobiotics^15^, or influences of the gut microbiome^16^.

Adipogenesis is a fairly well-characterized process and many of the key genes involved have been previously described in^11^,^17^,^18^. However, information on the coordinated, time dependent changes in pathways involved as well as a better understanding of the networks and interactions of the genes and pathways participating in the process of adipogenesis (*i.e.*, a system-level understanding) is crucial to better identify potential interventions and drug targets in treating and preventing obesity and its co-morbidities. While network-based analysis of genes has been more recently explored to better understand adipocyte differentiation in 3T3L1 adipocytes of rodent origin^19^,^20^,^21^, such analyses are lacking in human adipocytes.

We propose a novel method (called network activity score finder or NASFinder) that integrates omics data with network analysis and tissue specificity information and we use it to improve our understanding of adipocyte differentiation in human adipocyte. Network analysis can be seen as a way of integrating new experimental evidence with previous knowledge about biological systems: the novelty of NASFinder is in the way it addresses the tradeoffs inherent in this type of analysis. In its simplest form, network analysis first seeks to expand a list of differentially expressed genes/proteins/metabolites resulting from the experiment at hand with the help of known molecular interactions, and then tries to identify the biological context of the enlarged list using functional annotations in the form of gene ontologies and canonical pathways. The two most critical steps of the whole workflow remain the expansion of the initial gene list and the identification of the relevant functional context both of which demand a careful balance directly affecting the overall sensitivity and accuracy of the analysis. Several approaches have been published that differ in how they address these critical points. EnrichNet^22^ produces a ranking of reference datasets based on a random walk algorithm that scores the distance between the input genes and the collection of reference gene sets; the same reference datasets can also be scored using tissue-specific association scores computed for 60 human tissues to further guide the search. DRAGEN^23^ is designed to identify differentially regulated reference gene sets by analyzing how the strength of interaction between all TF-target pairs included in each gene set changes between experimental conditions (*e.g.*, between control and treated samples), and estimating the significance of such changes. SPIA^24^ introduces a type of network analysis specifically formulated for signaling pathways and combines traditional enrichment analysis with a score that measures the perturbation of a pathway under a given experimental condition.

NASFinder implements a number of strategies of network exploration that try to reproduce the advantages of these methods without some of their limitations, such as the need to have a directed background network as in SPIA or network annotations in terms of TF-target pairs as in DRAGEN. First, NASFinder constrains the initial expansion of the gene list by performing a guided exploration of the interaction network which must include at least one ‘source’ node at the upstream end, such as a receptor, transporter, or transcription factor (the specific member of the user-defined class of molecules is automatically selected). Second, the exploration is further constrained by restricting it to a user-specified tissue-specific network. The use of tissue-specific networks is an advantage over the post-analysis association scoring based on tissue-specific gene expression profiles used by EnrichNet because we do not consider at all interactions not specific to the tissue. Additionally, our method ranks the enriched sub-networks identified in the guided exploration according to their activity level in the experimental data sets relying on an information flow algorithm. Conceptually, we follow a systems biology interpretation of cellular processes where biological components are considered as information processing units and their interactions as information exchange steps^25^,^26^,^27^,^28^, effectively employing this abstraction as a guide for the identification of relevant functional contexts. The sub-networks can then be functionally annotated in terms of canonical pathways (*e.g.*, Reactome database) or gene ontologies. We also developed a set of novel visualization tools to illustrate the mapping of data into biological context and to enable enhanced biological interpretation. The complete pipeline is illustrated in Fig. 1A. NASFinder is available at the URL http://www.cosbi.eu/research/prototypes/nasfinder as a free web service.

To elucidate the key mechanisms, and time dependent changes in the interacting networks and pathways driving human adipocyte differentiation, we used human Simpson-Golabi-Behmel Syndrome (SGBS) euploid progenitor cell that differentiates to adipocytes in culture^5^,^29^,^30^. Microarray-based transcriptional profiling was conducted at different stages of differentiation *in vitro*. RNA samples from biological triplicates were analyzed at 0, 6, 48, 96, 192, and 384 hours after induction of adipogenesis. The main analyses we performed were the comparison of each time point with the control (0 hours), and comparison of each time point with the previous one. The combination of the two provides a clearer picture of the activation and de-activation of the processes and their relationships that are fundamental to sustain the complete differentiation of pre-adipocytes into mature adipocytes. The data from the samples, our novel method, and the results of the analyses were used for building the first systems view of human adipocyte differentiation.

## Material and Methods

### SGBS Cell culture

Human Simpson-Golabi-Behmel syndrome (SGBS) preadipocytes that were provided by Martin Wabitsch were used in this study and cultured as described previously^31^. Briefly, the SGBS preadipocytes cells were cultured at 37 °C in a humidified incubator maintaining a 5% CO_2_ atmosphere. The growth medium consisted of DMEM:F12 (1:1) (GIBCO, Life technologies, Grand Island, NY), 33 mM biotin, and 17 mM pantothenate containing 10% fetal bovine serum (Hyclone, Logan, UT) and 1% penicillin-streptomycin (GIBCO, Life technologies). For this study, SGBS preadipocytes were plated at 1 × 10^5^ cells per well of a 6 well plate. The cells were grown to confluence and induced to differentiate into adipocytes one day post confluence by addition of a serum-free differentiation medium. The differentiation medium consisted of DMEM:F12 (1:1) (obtained by mixing Dulbecco’s Modified Eagle’s Medium without glucose (SIGMA) and Hams F12 nutrient mixture containing 10 mM glucose (SIGMA) in a 1:1 ratio) to which 25 nM dexamethasone, 500 μM 3-isobutyl-1-methylxanthine, 2 μM rosiglitazone, 0.01 mg/ml human transferrin, 2 × 10^−8^ M insulin, 10^−7^ M cortisol, 0.2 nM T3, 33 mM biotin, and 17 mM pantothenate was added. The final glucose concentration in the medium was 5 mM glucose, equivalent to the normal blood glucose concentration. The cells were maintained in differentiation medium for 4 days after which the medium was changed to a serum-free adipogenic medium consisting of DMEM:F12 (1:1) with 0.01 mg/ml human transferrin, 2 × 10^−8^ M insulin, 10^−7^ M cortisol, 0.2 nM T3, 33 mM biotin, and 17 mM pantothenate. The adipogenic medium was essentially similar to the differentiation medium but without 3-isobutyl-1-methylxanthine (IBMX), dexamethasone and rosiglitazone. The medium was renewed every two days from the initiation of differentiation. Cells were harvested in triplicates for each specific time point including 0, 6, 48, 96, 192 and 384 hours, following the initiation of differentiation to examine the time-dependent changes in mRNA transcripts. During harvesting, the medium was aspirated and the cells were harvested using Lysis/Binding Solution (Ambion^®^, Life Technologies). Total RNA was isolated from the harvested cells using the RNAqueous^®^ Total RNA Isolation Kit (Ambion^®^, Life Technologies Inc., Carlsbad, CA) as per the manufacturer’s recommendations. The quality of the RNA isolated was examined using Agilent 2100 Bioanalyzer (Agilent technologies, Palo Alto, CA). RNA integrity numbers of >9 were obtained in each case. The quantity of RNA was measured using the NanoDrop 8000 (Thermo Scientific Wilmington, DE) and the 260/280 ratios obtained were between 1.8 and 2.1.

### Transcriptomics data

The dataset was generated with 8 Illumina Human HT-12 version 4 BeadChips (Ilumina, Inc., San Diego, CA) hybridized with the RNA from 18 cell-cultures at different time points (0, 6, 48, 96, 192, 384 hours). The RNA labeling and microarray hybridization was performed according to the manufacturer’s recommendations. The data file contains 26 arrays (including technical replicates)^31^ and is available on GEO (accession number GSE76131).

#### Normalization, Variance stabilization

A list of 19 variance stabilization/normalization methods were compared (Supplement Table 8 in Supplementary file 1) to select the best one for the dataset by assessing the relation between the empirical standard deviation and the rank of the mean expression for each method. When plotting the standard deviation versus the rank of the mean, an ideal method would produce a parallel line to the x-axis where some random fluctuations might exist but without exhibiting an overall trend. The plots for all the methods (Supplement Fig. 5 in Supplementary file 1) were created using the diagnostic functions available in the VSN R package^32^. According to the above criteria, the VSN method was selected for calibration and variance stabilization of the data.

#### Filtering

Filtering out irrelevant or noisy data helps in reducing the burden of multiple testing and thereby improves the power to detect differential expression. Probe sequences were annotated with four quality categories (‘Perfect’, ‘Good’, ‘Bad’ and ‘No match’ - Supplement Fig. 6 in Supplementary file 1)^33^. The definition of the quality categories is: *Perfect* if it perfectly and uniquely matches the target transcript; *Good* if the probe is still likely to provide considerably sensitive signal, even though it imperfectly matches the target transcript; *Bad* if the probe matches repeat sequences, intergenic or intronic regions, or is unlikely to provide specific signal for any transcript. *No match* if it does not match any genomic region or transcript. Probes classified as either ‘Bad’ or ‘No match’ (illuminaHumanv4.db in R) were removed as represented in red in the histogram in Supplement Fig. 6 in Supplementary file 1.

#### Re-annotation

The Illumina Human HT-12 version 4 data were re-annotated (illuminaHumanv4.db in R) to avoid some inconsistencies of the data set. For the missing EntrezID mappings an additional annotation script was implemented in R that works also for non-Illumina data. Gene symbols (HUGO and non-official synonyms) were converted into EntrezID by analyzing the consensus results from Ensemble (via web service) and the Genome wide annotation for Human (org.Hs.eg.db in R) [The HUGO and non-official gene name is associated with the EntrezId as determined by the intersection of results from Ensemble and org.Hs.eg.db. If not empty, then the merge of what was produced by org.Hs.eg.db was used where more than one EntrezId can be associated with the same gene name.]. These steps increased the ratio of significant gene markers usable in public DBs from 65–80% of previous workflows up to 99% of recognized gene symbols. Downstream enrichment and pathway analyses can now benefit from 99% of the overall significant biological signals detected by the differential expression analysis.

Statistical empirical array quality analysis as implemented in limma^34^ was applied to associate quality weights with all arrays. The computed array quality weights were used in the successive identification of the differentially expressed genes. To take the correlation structure between the technical replicates into account we used the *duplicate Correlation* function in limma.

### NASFinder pipeline

**Figure 1:**
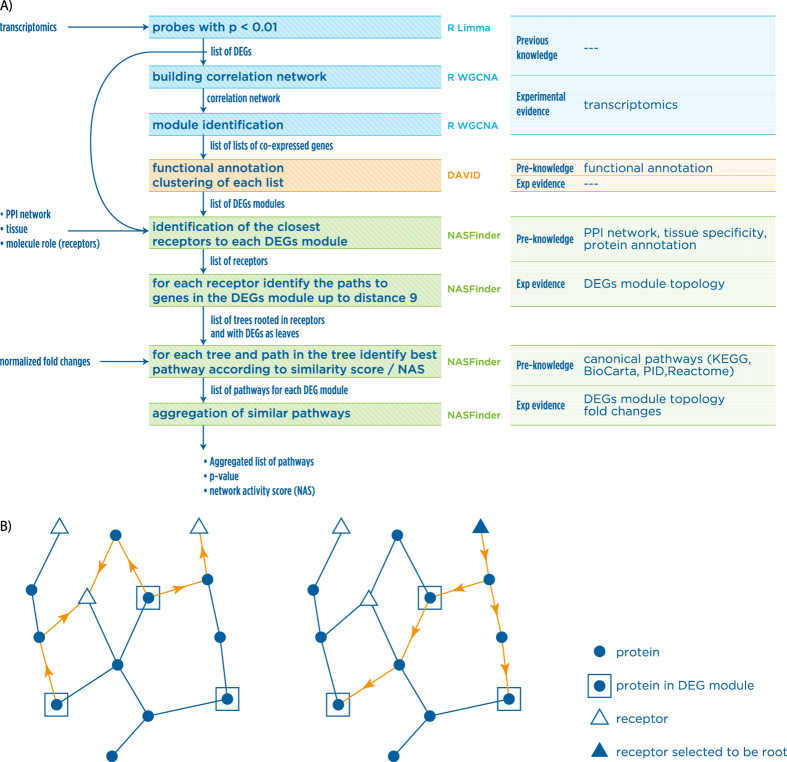
(**A**) NASFinder pipeline. The input is a transcriptomic data set used to detect sets of genes that are differentially expressed and that have common biological functions (DEGs modules), a set of nodes of interest with specific functions (transporters, transcription factors, receptors, etc.), and a tissue-specific reference network to contextualize the gene sets for a better interpretation. The algorithm determines active sub-networks connecting receptors with DEGs modules and ranks them according to the network activity score. The significant sub-networks are then used for contextual enrichment analysis against canonical pathways. (**B**) Sub-network identification. The shortest paths from each element of the DEG module to the receptors are computed and the shortest ones are kept. In the figure the orange paths are the ones with minimal distance greater than 0 from the DEGs module to the regulator molecules (receptors in this study). In the next step (right graph) for each receptor previously selected (for instance, the top-right receptor in this case) we identify all the shortest paths from that receptor to the elements of the DEGs module (the orange paths).

The novel NASFinder method is introduced in the Results section (and illustrated in Fig. 1A) because it is a main achievement of this study. The inputs of the pipeline are a set of functionally related and differentially expressed genes, an human interactome network and a class of molecules tagged as main regulator for the ongoing study (receptors, transporters, etc.). NASFinder applies an information flow algorithm to detect the most active sub-networks connecting the main regulators and the genes in the input module. The next-subsections briefly discuss the methods underlying the computational process of scoring the activity level of network functional modules.

### Identification of significantly differentially expressed genes

This sub-section refers to the first box in the pipeline reported in Fig. 1A. After data filtering and normalization, the set of differentially expressed probes were identified and used in the first step of the NASFinder pipeline. When multiple high-quality DE probes were available per gene, we selected the most significant (the lowest p-value). The outcome of each analysis was a list of differentially expressed genes (DEG) that are then grouped in clusters of functionally related genes (DEG modules, see next sub-section).

#### Time-point differential expression analysis of controls

This analysis identified the differentially expressed genes at each time point 6, 48, 96, 192, 384 hours with respect to the time point 0 h, *i.e.* controls at each time point were compared with the baseline at time 0 h. The analyses were performed using the limma^35^ R package and the probes were ranked by their log-odds scores given by empirical Bayesian moderation of sample variances and a FDR threshold set at 0.01. The results of these analyses are summarized in the Venn diagram and histogram in Supplement Fig. 3 in Supplementary file 1 (see also Supplementary file 2-S3).

#### Time-lapse differential expression analysis of controls

Time-lapse analysis identified the differentially expressed genes for each two consecutive time points (6 vs 0, 48 vs 6, 96 vs 48, 192 vs 96, 384 vs 192 hours). The time-lapse analysis detects what changes specifically at each time interval; which differs from the time-point analysis that identified changes between a time point and baseline. Differentially expressed probes were determined by using the moderated t-statistic with empirical Bayesian shrinking of variances and a FDR threshold set at 0.01. The results of these analyses are summarized in the Venn diagram and histogram in Supplement Fig. 4 in Supplementary file 1 (see also Supplementary file 2-S3).

### Correlation analysis and identification of DEG modules

This sub-section refers to the second, third and fourth boxes in the pipeline reported in Fig. 1A. An R pipeline was developed to identify co-expressed and functionally related gene sets (hereafter DEG modules). This pipeline relies on the external web-services Ensembl and DAVID. We first outline the steps in the construction of gene co-expression lists (Supplementary file 2-S4), then we describe the functional annotation of TP and TL analyses.

#### Co-expression modules

The differentially expressed probes identified in time-point and time-lapse analyses were used to construct the weighted gene co-expression modules using the R package WGCNA^36^. The bi-weight mid-correlation was chosen because it is more robust to outliers and varying conditions than Pearson’s correlation^37^. The dynamic branch cutting algorithm from the same package was used to detect the modules of co-expressed genes and a WGCNA co-expression analysis was run for each time-point and time-lapse comparison. A parameter scan-based selection of the three main parameters (correlations sign, power 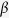 and sensitivity to cluster splitting in the dynamic tree cut) was carried out by manually inspecting the quality of the resulting co-expression clustering dendograms (in terms of definition and noise) to obtain coherent and compact clusters of co-expressed genes (Supplementary file 2-S4).

#### Identification of functionally related gene sets (DEG modules)

Each cluster of co-expressed genes contained a large number of genes. Therefore the clusters were decomposed according to the functional categories of their genes related to biological functions and pathways (DEG modules). The EntrezIDs for the genes of each cluster were functionally grouped with DAVID v6.7 and the kappa statistic (determined within DAVID) with the default parameters^38^. The following annotation databases were used for functional annotation clustering: KEGG, BIOCARTA, REACTOME, PANTHER, GO, MINT, INTERPRO, SP_PIR_KEYWORDS, PIR_SUPERFAMILY, UP_SEQ_FEATURE, COG_ONTOLOGY, BBID and SMART. Analyses and results were managed from within R workspace via web services using the R DAVID Web Service package v1.2^39^. All functionally clustered groups with DAVID enrichment score greater than 1 were considered (DAVID’s enrichment score 1.3 is equivalent to 0.05 p-value, however our automated pipeline allowed us to analyze many potentially interesting groups at a slightly lower threshold than recommended by DAVID’s authors^39^). However, only the genes associated with functional terms whose a p-value was smaller than 0.05 were considered to form a DEG module (Supplementary file 2-S5). The workflow was automated for handling genome wide analysis. The outcome of this step was a list of DEG modules of genes that are co-expressed and functionally related in biological functions computed for each time-point and time-lapse analysis. NASFinder considers a DEG module at a time and can be iteratively applied to all the DEG modules detected.

### Preparation of the human interactome for network analysis and main regulators selection

This sub-section refers to the fifth box in the pipeline reported in Fig. 1A. The default interactome that NASFinder used is the largest manually curated human signaling network and was created by the Wang Lab (http://www.cancer-systemsbiology.org/dataandsoftware.htm). The network consists of 6000 proteins and 63000 interactions determined from review literature on cell signaling and other sources including BioCarta, CST Signaling pathways, Pathway Interaction database (PID), and Information Hyperlinked over Proteins (iHOP). The Wang Lab network is updated yearly (for this study we used version 6 released in 2014). This interaction network is referred to as reference network in the following.

#### Expanding the reference network

A main step of NASFinder is mapping a DEG module to the reference network. However, some genes in the module were not in the reference network. To improve the quality of the analysis, NASFinder accepts nodes and interactions to be added to the reference network as an additional input data set from the user. Finally, NASFinder expands the reference network by adding the missing genes and interactions from BioGRID^40^. The resulting reference network has a much better coverage of the genes in the DEG module.

#### Tissue specificity

The user may specify a reference tissue to perform the analysis. In this case, NASFinder prunes the expanded reference network to reflect tissue specificity relying on the tissue-based map of the human proteome^8^ (www.proteinatlas.org/humanproteome/tissue+ specific). The map is composed of 32 human tissues and classifies genes in terms of tissue specificity according to the following three definitions:

1. tissue enriched genes (TE): genes with at least 5-fold greater expression (expressed as fragments per kilobase of exon per million mapped reads; FPKM) in one specific tissue compared to all others,
2. group enriched genes (GE): genes with at least 5-fold greater expression in a limited number of tissues compared to all others,
3. tissue enhanced genes (TEn): genes that do not fulfill the criteria of tissue enriched but show a 5-fold higher level in a specific tissue type compared to the average FPKM value of all 32 analyzed tissue types.

NASFinder implements tissue specificity by pruning from the expanded reference network all the tissue enriched and group enriched genes for the tissues that are different from the selected one without removing the genes that are tissue enriched, group enriched, or tissue enhanced for the selected tissue.

#### Selecting the closest regulators to the DEGs module

In order to help focus the analysis of specific biological questions while computing the network activity score and the functional annotation of the sub-network modules detected, the user defines a class of molecules with a specific biological role (*e.g.*, receptors, transporters, transcription factors, etc.). The outcome of this selection is an increased sensitivity of the results of NASFinder. The type of molecules is defined according to the annotation of the initial reference network. The user can expand the list of molecules of a chosen type by providing an additional annotated network as input to NASFinder. The list of typed molecules does not change when expanding the reference network with BioGRID nodes and interactions. This study used receptors as candidate regulators (Supplementary file 2-S6) since this class included transcription factors (nuclear receptors), membrane, and other types of signaling receptors. Members of this “family” have been shown to be involved in adipogenesis. However, a tenet of systems analysis is to not restrict inputs to only those genes/proteins previously shown to be involved in a given biological process (in this case, adipogenesis). Including all members of this class provides an opportunity to discover unsuspected pathways and networks.

To keep the signaling function of receptors focused on the experimental conditions (implicitly encoded in the DEGs module), we selected all the closest receptors to the DE genes of the module (minimal distance greater than 0) as main regulators plus the receptors differentially expressed and members of the DEGs module (Fig. 1B). The selection is implemented by a breadth-first search to select the shortest paths from the molecules in the DEG module to candidate regulators (in this study receptors). Note that this step takes advantage of the experimental evidence encoded in the DEGs module and of previous knowledge encoded in the topology of the reference network.

### Contextual enrichment analysis and network activity score

This sub-section refers to the sixth, seventh, and eighth boxes in the pipeline reported in Fig. 1A. Edges of the network were labeled with a common linkage index that uses common neighbors as an evidence of the strength of the interaction between two molecules^3^,^41^. Consider two nodes *a*_*i*_ and 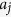 with a direct interaction value in the adjacency matrix *D*_*ij*_ and define *I*_*ij*_ as the number of common neighbors of the two nodes (representing the indirect interactions between *a*_*i*_ and *a*_*j*_). The common linkage index (CL) used to label the edge between *a*_*i*_ and *a*_*j*_ is


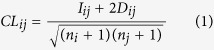


where *n*_*k*_ is the number of neighbors of the node *a*_*k*_. The common linkage index is a proportion of the common shared neighbors with respect to the total neighbors of the two nodes *a*_*i*_ and *a*_*j*_. We assume that the common linkage index between two nodes that are not connected is 0.

For each key regulator identified in the previous section, NASFinder traverses the network by retaining the path from the regulator to each gene in the DEG module that has the highest score computed by multiplying the *CL*_*ij*_ of each edge in the path^42^. NASFinder subsequently generates a network by merging all the paths (up to length 9) retained in the traversal of the graph and then, optionally, extends it to include the 1-neighbor of each node. The outcome of this step is a set of minimal sub-networks, one per DEG module, that include most of the genes in the input DEG module (those at distance <10 from at least one key regulator). A cap of 10 on distances was set because longer paths are rare and unlikely to bring additional benefits.

NASFinder uses the genes in the DEG module that belong to the minimal network identified in the previous step as target set *T*. NASFinder also uses reference gene sets *R*_1_, …, *R*_*m*_ representing the canonical pathways as defined by 4 reference databases (KEGG, BioCarta, PID and Reactome)^43^. NASFinder computes the enrichment score of the common molecules between the target set 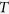 and the genes in the canonical pathways *R*_*i*_ by using the *Sorensen-Dice Similarity index:*


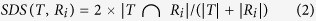


where |*T*| and |*R*_*i*_| are number of molecules in 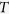 and 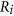, respectively. The 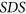 ranges between 0 and 1, and accounts for the over representation of small gene sets^44^,^45^. A p-value associated to the overlap between the target set 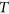 and the genes in the canonical pathways 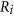 is computed using the hypergeometric distribution; no multiple testing correction is applied because these p-values are used only for ranking purposes. NASFinder repeats this analysis for all minimal networks and for all reference gene sets, calculates the *p*-values and selects the most relevant reference set (the lowest *p*-value).

#### Network activity score and module ranking

NASFinder also computes a score of the activity of the identified network called network activity score (NAS). NAS was designed to take into account both (i) the activity of the genes that are both in the DEG module and the identified sub-network, and (ii) the proportion of genes that are differentially expressed over the total of pathway genes. NAS is used to evaluate the impact of networks for a given experiment by utilizing corresponding variation to omics data^46^,^47^. NAS is based on the number of common molecules between the experimental data and the selected reference signature (CDR), the number of molecules in reference signature (NGR) and the mean of normalized fold change of CDR (MNF). NAS = (MNF × CDR)/NGR is used to represent how the selected reference gene signature was influenced by the experimental conditions, based on the magnitude of fold changes (*e.g.*, concentration or intensity level)^48^. The normalization of the fold change is done with a scaling method that maps fold changes into the interval [0, 1]. Specifically, a fold change *v* is mapped to


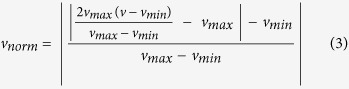


where *v*_*max*_ and *v*_*min*_ are the maximum and minimum fold changes taken with sign. Finally, each node of the reference network corresponding to genes in the DEG module is labeled with its normalized fold change. We found this normalization method to outperform other normalization strategies in our study of pre-adipocyte differentiation because it favors positive fold changes that represent activation of pathways in the differentiation process.

## Results

The main results of this paper are a new method (NASFinder) for identification and activity ranking of the main biological processes using omics data and its specific application to gene expression data generating the first systems view of the adipocyte differentiation process in culture. The following sub-sections describe these two aspects and also provide a comparison of the NASFinder method against other network analysis pipelines using publicly available transcriptomic data sets. A more complete description of the methodology and biological results from SGBS cells are reported in Supplementary file 1. We start describing the novel method and then we move to the systems view of adipogenesis by interpreting the results of NASFinder.

### A novel pipeline to detect and rank tissue-specific sub-networks identified by omics data

We developed a new method (NASFinder) to identify tissue-specific sub-networks connecting an omics-determined module to its main regulator(s) chosen among a user-defined class of molecules (*e.g.*, receptors, transcription factors, etc.). The information flow from differentially expressed genes (DEG) to the main regulator (nodes) in the network topology is used to associate nodes with an activity score and ultimately to determine the sub-network activity score (NAS index). Finally, the sub-network is annotated using comparisons to canonical pathways or gene ontologies to predict its biological function. The pipeline that we developed and adopted in the paper is schematized in Fig. 1A and is made up of the following logical steps:

1. *Transcriptomic module identification*. Pre-processing of transcriptomic data and identification of sets of differentially expressed genes that are co-expressed and that have similar biological functions (DEGs modules). This step is performed in the first four blocks in Fig. 1A relying on the R packages Limma and WGCNA and finally applying DAVID for a preliminary functional annotation.
2. *Identification of the main regulator molecules*. Each DEGs module is mapped onto the largest manually curated human signaling network (Wang Lab - http://www.cancer-systemsbiology.org/dataandsoftware.htm), our reference network, consisting of 6000 proteins and 63000 interactions determined from review of the literature on cell signaling and other sources including BioCarta, CST Signaling pathways, Pathway Interaction database (PID), and information hyperlinked over proteins (iHOP). The network is updated yearly (ver. 6 - 2014 was used in this study). The network was expanded with genes in the DEG module and their interactions that were not in the reference network, but are available in BioGRID. After selection of the user-defined class of regulator molecules, the network was pruned according to tissue specificity. Through an iterated application of the breadth-first search algorithm starting from DEGs of the module, we identified the closest regulator molecules to the DEGs module (distance greater than 0). We then extended the set of regulators to include all the differentially expressed regulators within the module (fifth box in Fig. 1A and left graph in Fig. 1B).
3. *Contextual enrichment analysis.* For each closest regulator molecule of each DEGs module, the paths (up to length 9 – for each length the shortest path to DEG is selected) from the regulator to a DEG in the module are collected and ranked according to the NAS score. For all the selected paths, a sub-network is built by extending them with their 1-neighbors. The sub-networks undergo an enrichment analysis using canonical pathways from the databases KEGG, BioCarta, PID and Reactome. For each receptor, the sub-network with the highest similarity score with a canonical pathway is chosen. If multiple sub-networks share the same similarity score, the sub-network with the greatest NAS is selected. Finally, similar pathways determined in the enrichment analysis are aggregated for ease of interpretation.

Steps 2 and 3 are repeated for each DEG module identified in step 1. The added value of NASFinder with respect to similar methods is its ability of managing tissue specific or cell specific networks as well as the ability to select molecules with a specific role (*e.g.*, receptors or transcription factors etc.) that will enable a very broad or a highly specific and focused interrogation of the data as needed.

### Visualization

**Figure 2:**
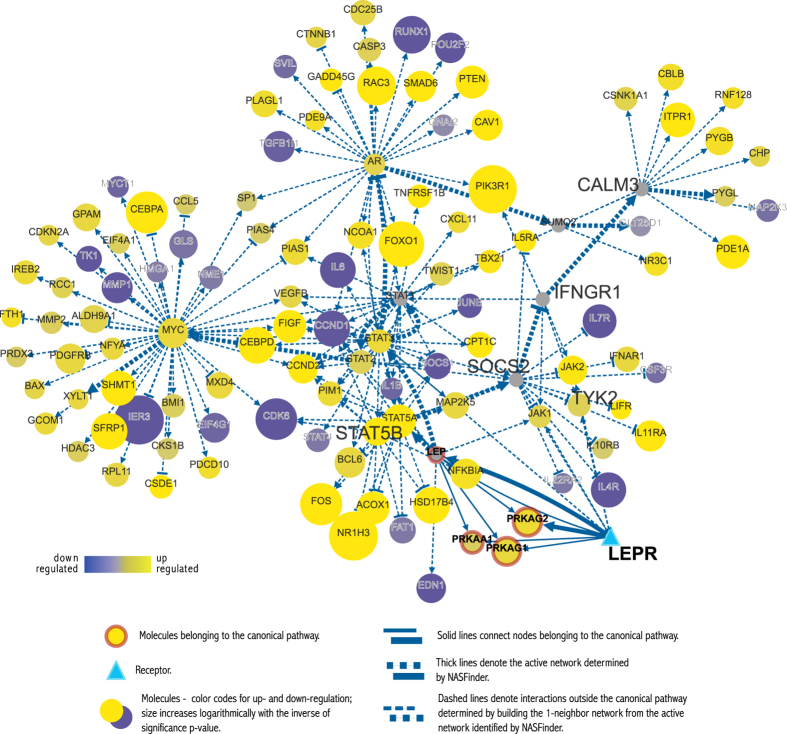
Leptin pathway and contextual interactions identified for contrast 48h vs. controls. LEPR is the receptor and is the entry point for identifying the active sub-network determined by NASFinder. The molecules belonging to the canonical pathway and within the 1-neighbor network of the active network determined by NASFinder are *LEPR*, *PRKAG2, PRKAA1, PRKAG1*.

An integrative visualization approach was used to summarize and visually combine in a common frame multiple analytical results coming from the NASFinder pipeline. The main outcome is the set of canonical pathways predicted by NASFinder. NASFinder identified the leptin receptor pathway, known to be up-regulated during adipocyte differentiation, which is shown in Fig. 2 as an example of the main NASFinder output. When multiple DEG modules resulted in the same active canonical pathway, the identified networks have been summarized together to offer a more comprehensive view of the interactions around the same active pathway. To visually combine topological and analytical information, we produced graphical output in different formats, including XGMML and interactive visualizations with force-directed layout^49^,^50^,^51^ (Supplementary file 3 - http://www.cosbi.eu/3867/NASFinder_Supplementary_file_3.zip - tested only on Firefox).

Genes often belong to one or more pathways at a given time point or in a given comparison. To capture this component of systems networks, we used a crosstab table in EXCEL for all DEG at a time point and all NASFinder pathways (Supplementary file 2-S2) and we represented it graphically in Circos visualizations (Supplementary file 3 – available at the URL http://www.cosbi.eu/3867/NASFinder_Supplementary_file_3.zip).

### Benchmarking NASFinder performance

We compared NASFinder with 15 representative tools^22^,^23^,^53^,^54^,^55^,^56^,^57^,^58^ performing overrepresentation analysis (ORA) and quantitative enrichment analysis (QEA) on 10 data sets. ORA and QEA are gene set enrichment analysis approaches that are used to functionally annotate a list of genes of interest^22^,^59^. A gene list is the only requirement for performing ORA whereas QEA uses abundance or differential expression levels to weight each gene. Some existing ORA and QEA tools map genes onto interaction networks and use network topological properties to improve the result of enrichment analysis^23^,^48^,^59^,^60^.

The data sets that we used as benchmark are the expression data from 10 cancer cell lines (Supplementary file 2-S1) which are accessible on GEO (accession numbers GSE24065, GSE11352, and GSE18684^61^,^62^,^63^,^64^). To compute true positive, true negative, false positive and false negative predictions of the tools we annotated all reference pathways collected from KEGG, Reactome, PID and BioCarta as positive or negative for each experimental setting. The pathways related to TP53, NFkB and ER genes were selected as positive results (for instance, pathways containing TP53 were annotated as positive in experiments where the treatment was Doxorubicin). For the LNCaP data set (GSE18684), the pathways including the androgene receptor (AR) were selected as positive results. Supplementary file 2-S1 contains the description of all the conditions used to determine the positive results as well as the annotations of all the reference pathways used in the benchmark.

The comparison was performed by running all of the tools on the data sets described above with default parameters and using the annotated reference pathways to define positive and negative results. The raw output results for all the tools are reported in Supplementary file 5 (available at the URL http://www.cosbi.eu/3867/NASFinder_Supplementary_file_5.zip). Some of the tools only use a subset of our reference DBs and for them we used as annotated references the corresponding subset of the table in Supplementary file 2-S1. We defined true positive (tp) a positive pathway (according to the manual annotation) with p-value less than 0.05, true negative (tn) a pathway annotated as negative and with p-value greater then 0.05, false positive (fp) a negative pathway with p-value less than 0.05 and false negative (fn) a positive pathway with p-value greater than 0.05. The standard measures we used to compare the tools are:

1. *Precision* defined as tp/(tp + fp)
2. *Recall or Sensitivity* defined as tp/(tp + fn) = tp/positives
3. *Specificity* defined as tn/(tn + fp) = tn/negatives
4. *Accuracy* defined as (tp + tn)/(positives + negatives).

**Figure 3:**
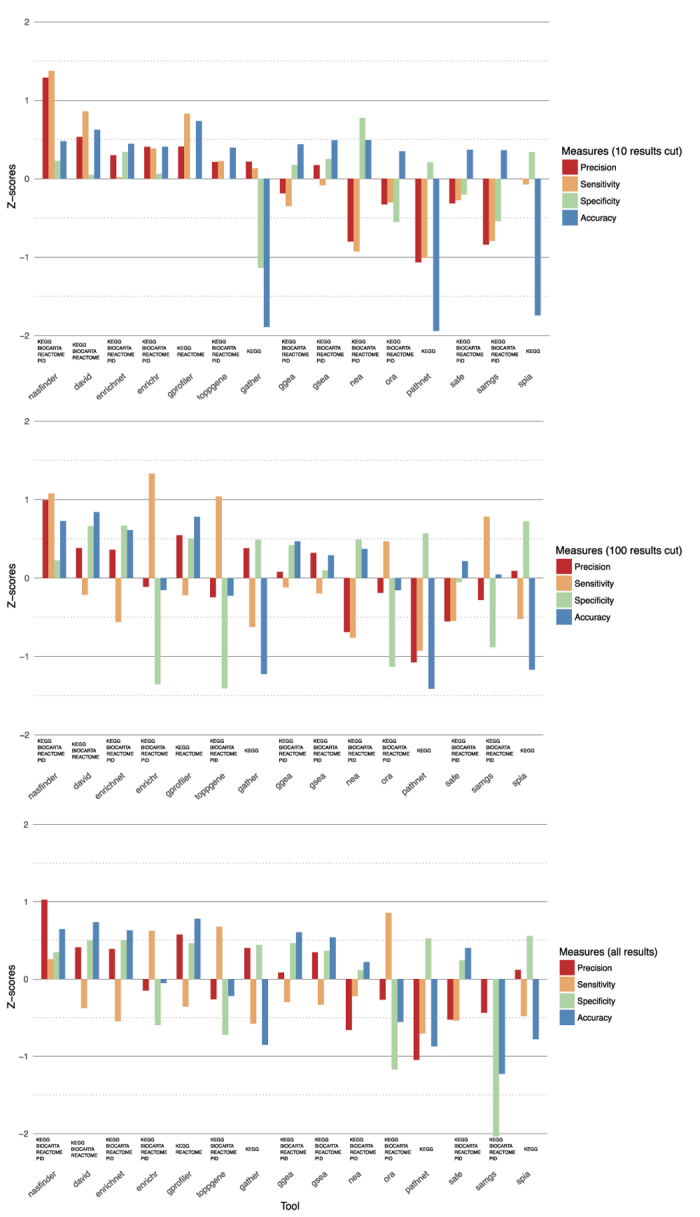
The performance assessment of the tools shown on the y-axis, based on z-scores of precision, recall, specificity, accuracy computed on the 10 benchmark data sets. NASFinder is the only tool with scores above the average in all three scenarios (i.e. the z-scores of all the performance measures are positive in all scenarios).

**Figure 4:**
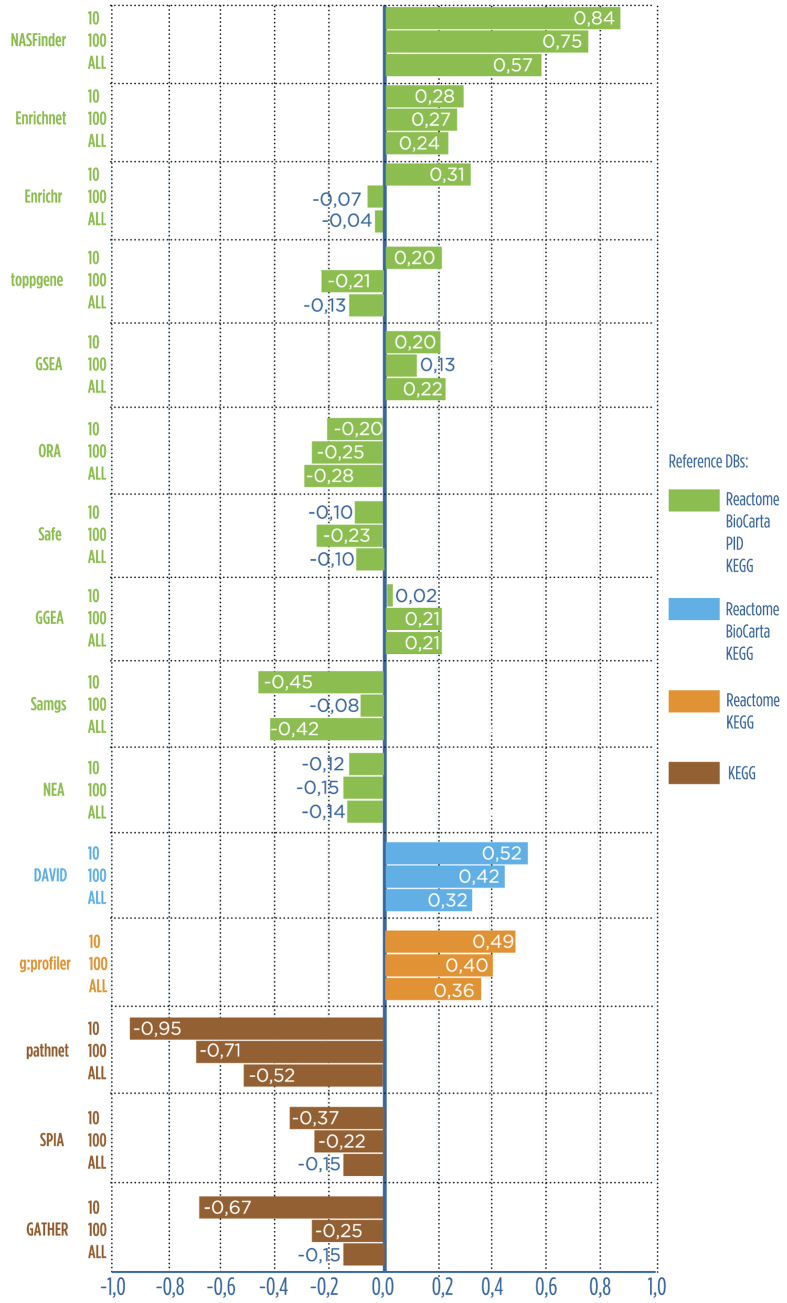
The overall performance of the tools shown on the x-axis expressed in terms of the average of the z-scores computed for precision, recall, accuracy and specificity on the 10 benchmark data sets. Colors of bars identify the reference databases used to compute the performance measures. NASFinder outperforms all the other tools in terms of aggregate performance.

We considered 3 scenarios: the 10 top-ranked results, the 100 top-ranked results and all results from each tool in order to avoid bias due to the choice of the cut. It is difficult to compare the tools by considering only the standard measures mentioned above because no tool outperforms the others in all the measures, although NASFinder is the only tool that always performs better than average with positive z-scores in all 3 scenarios (Fig. 3 and Supplementary file 2-S1, respectively). We then considered the average of the z-scores computed on each standard measure as a cumulative quality measure. NASFinder outperformed all the considered tools using this aggregate measure of performance (Fig. 4). We attribute this better performance of NASFinder to the combined use of tissue specificity, network exploration strategy, and network activity scoring.

### Curation of adipocyte differentiation analyses –A systems view

NASFinder provided a more comprehensive analysis of the global transcriptional state of human SBGS cell line *in vitro* during adipogenesis than most published reports by identifying the most active sub-networks at 6, 48, 96, 192 and 384 hours compared to the undifferentiated cell. This euploid progenitor pre-adipocyte is now a widely used human model of adipogenesis^30^,^65^ and has been shown to be comparable to the aneuploid (2n = 40) mouse 3T3-L1 pre-adipocytes^65^,^66^. The neutral lipid stain, Oil Red O was used to assess lipid accumulation in the adipocytes used in this study at different time points (0, 48, 96, 192 and 384 h) from the induction of differentiation at 0 h (Supplement Fig. 1 in Supplementary file 1). Time point analysis (TP) consisted of identifying networks of differentially expressed genes (DEG) determined as the ratio of expression at each of 5 time points (6, 48, 96, 192, 384 hr) compared to control (0 hour), while the ratio of expression at each time point compared to the previous time point constituted the time lapse analysis (TL). A total of 5 sets of data were produced for TP analysis and 5 for TL (Supplementary file 4). These data sets together provided a comprehensive analysis of the differentiation process viewed through sub-networks based on receptor nodes. Similar analyses were performed for transcription factors and transporters (not shown). Since cellular processes are by nature interacting systems, many of the functions identified in the pathways overlapped in TP and TL analyses. More detailed analyses of selected processes, including genes within these pathways with published references, are presented in Supplementary file 1.

Hierarchical analysis indicated that the majority of the pathways at each time point or between time points did not overlap (not shown), although some individual genes participated in multiple pathways (summarized in TP and TL Supplementary file 2-S2) and many pathways shared interactions when expanding the identified networks with 1-neighborhood nodes and interactions (Supplementary file 3 – http://www.cosbi.eu/3867/NASFinder_Supplementary_file_3.zip). In addition, NASFinder identified all paths between one or more source nodes (e.g., receptor) and a DEG module; this may generate multiple instances of the same canonical pathway. For example, three CARDIACEGF_Pathways were identified at 6 hours after induction with *EGFR*, *IL12RB2*, and *PPARA* as source nodes (see Supplementary file 2-S7, 6 v control). In other cases, multiple instances of a pathway with the same source node were identified. These results were expected since transcriptional regulation and resulting cellular processes consist of interconnected sub-systems. In some cases, we combine all pathways with the same name under the term, consolidated pathway.

**Figure 5:**
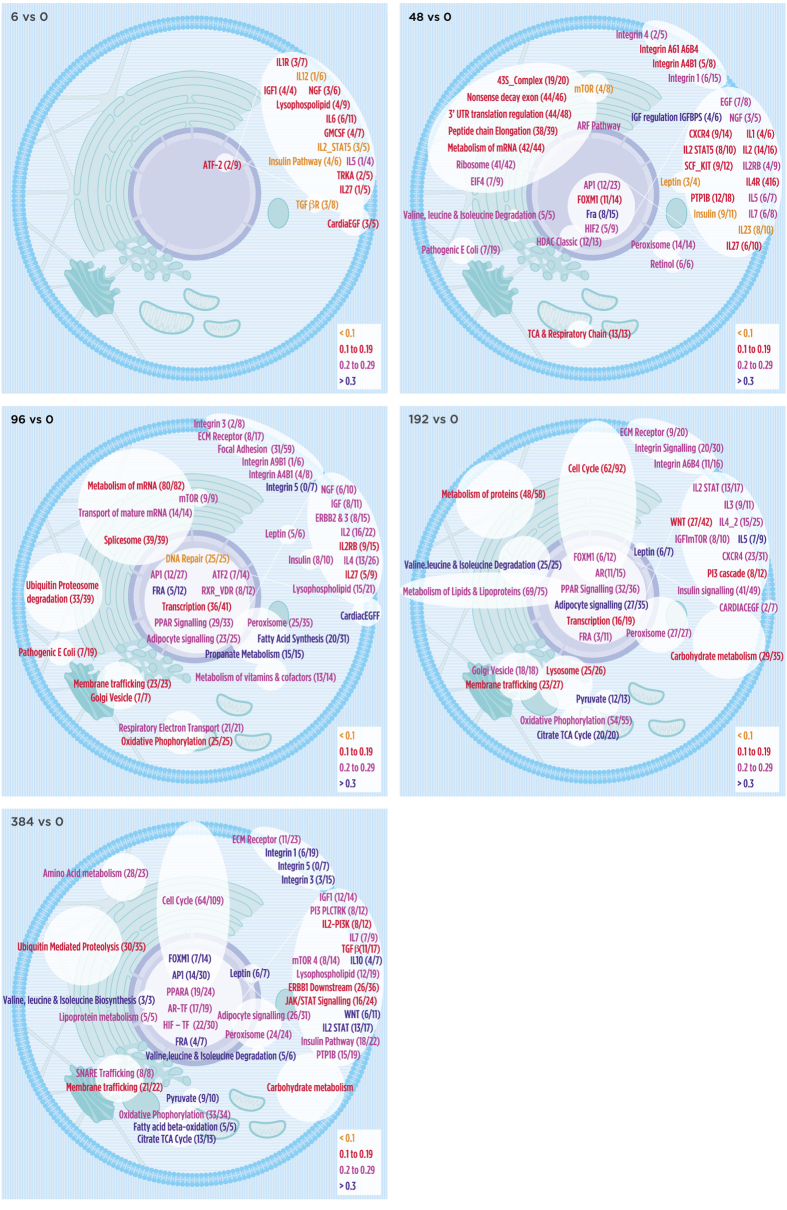
Selected pathways identified in the analyses are overlaid onto the cell based on the approximate location of the main cellular process of the genes involved and grouped by function. Note that many pathways and networks overlap cellular compartments which could not be represented in this format. The white lines connecting the signaling pathways to the nuclear pathways are used to indicate that these networks have components from the cell membrane to transcriptional machinery. The colors represent the network activity score of up/down-regulated differentially expressed genes traversed in the active path and its 1-neighborhood context. That proportion is reported as a fraction in parenthesis (x/y) denoting the number of up-regulated genes with respect to the total traversed and contextual ones. TP analysis.

**Figure 6:**
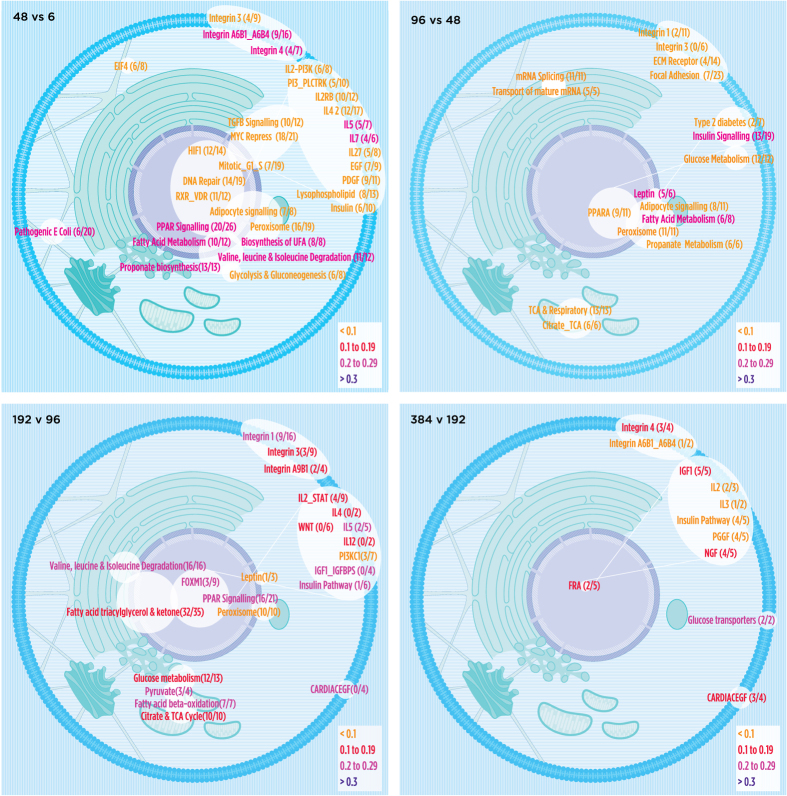
Same as Fig. 5, but for TL analysis.

A subset of pathways (usually NAS > 0.1 with p value < 0.05) for TP or TL is summarized visually using cell modules (Figs 5 and 6). For exact overlaps and network activity scores of all pathways – time point and time lapse – see Supplementary file 2-S2. The molecular pathways identified (Figs 5 and 6 and Supplementary file 1) can contribute to a better understanding of the molecular mechanisms involved in the adipocyte differentiation process.

### Time Point Analysis

The differences in abundance of genes at each time point versus pre-induction (0 hr) identified the networks involved in converting a pre-adipocyte to a mature adipocyte. The pathways identified were grouped by class to provide an overview of the differentiation process: signaling, transcription factor, metabolism, energy, structural membrane, and cellular structure (Table 1). Although the function of many of these pathways overlaps, especially signaling pathways that may be connected to cell membrane interactions and transcriptional regulation (white crossed lines in each figure), the table provides a summary of the relative activities of cellular processes at each time point. Signaling pathways are the predominant functional class at all time points with most of these pathways up-regulated, consistent with the selection of receptors as the source node. A diverse set of metabolic processes is induced across all time points but the majority is induced at 96, 192, and 384 hr. Examples of pathways identified by NASFinder at each time point are summarized in Table 2.

Forty-seven [47] consolidated networks were identified at 6 hours post induction, the majority of which had modest NASFinder scores (<0.1) relative to other time points. The state of the cell at this early differentiation point is best summarized as having up- or down regulation of many cytokine and intracellular signaling (Figs 5 and 6 vs 0). For example, CARDIACEGF regulates intracellular calcium concentration that contributes to the initiation of downstream signal transduction changes. Expression of cytokines, such as IL1 and IL6 in the IL1R pathway, can suppress adipogenesis^39^. Many cytokine pathways were down regulated at this time point (Figs 5 and 6 vs 0) and are very early events in facilitating induction and promotion of adipogenesis^67^.

The induction of insulin like growth factor activity, prostanoid ligand receptors (which can activate PPARs), translational machinery, cytoskeletal remodeling (pathogenic E. coli infection), insulin signaling, interconnected cell membrane structures, and transcriptional regulation were the most dominant pathways at 48 hours versus control (40 networks of 101 are shown in Fig. 5, 48 vs 0). These pathways are consistent with the increased protein requirement and cytoskeletal reorganization that enables a preadipocyte of fibroblastic morphology to switch into a more rotund cell that can load and accumulate lipid. Transcription of genes associated with central metabolic processes (triacylglycerol hydrolysis, branched chain and lysine amino acid degradation, TCA cycle, and porphyrin, pyruvate, retinol, galactose metabolism) was increased at 48 hours. Branched chain amino acid degradation may provide an alternative energy source for adipogenesis^68^.

The diversity in networks was most apparent at 96 hours when 145 consolidated pathways were identified. The 40 networks shown in Fig. 5, 96 vs 0, are representative of the utility of NASFinder: although genes overlap and connect subsets of pathways, a large number of networks were identified that affect different cellular processes involved in preparing the cell for production of adipocyte specific metabolic processes. For example, the three RNA pathways detected encompass ribosomal machinery, energy metabolism (oxidative phosphorylation, TCA cycle), the misidentified Alzheimer’s and Parkinson’s pathways (due to genes in the pathways initially shown to be associated with these diseases), and a more diverse set of metabolic networks were also induced at 96 hours, in addition to the bellwether adipogenic pathways, PPARγ/VDR and leptin.

Another set of pathways emerged at 192 (31 of 137 are shown Fig. 5, 192 vs 0) and 384 (40 of 137 networks are shown in Fig. 5, 384 vs 0) hours that can be considered adipocyte specific (PPAR_Signaling, peroxisome, leptin, insulin receptor signaling) or involved in maintaining the differentiated state (e.g. Cell_Cycle). As with other time points, over half the networks at each of these time points were involved in intracellular signaling. Although SGBS showed adipocyte characteristics by 8 days (our results and^65^), only 15 signaling pathways had high NAS at p < 0.05 were shared between 192 and 384 hours. A similar phenomenon occurred with metabolic pathways: only 8 pathways (folate biosynthesis, insulin pathway, glutathione, pyruvate, carbohydrate, nucleotides, pantothenate, and vitamins and cofactors) were in common between day 8 and 16. Many of these networks are also differentially expressed at the 384 vs 0 hour comparison, when the adipocyte is fully mature.

### Time lapse (TL)

Time lapse analysis identified changes between time points and provided a more dynamic view of the differentiation process (Fig. 6). The majority of pathways differentially regulated between time points (Table 3) involved signal transduction with their highest percentages of total pathways at the early (6 v 0 hr) and late (384 v 192 hr) intervals (91% and 72%, respectively). While many of the pathways that differed between time points were the same as those between a time point to 0 hr, the majority of these networks were further up-regulated as differentiation occurred (examples in Table 4). The intermediate time intervals had a greater number of pathways at p < 0.05 which were also more functionally diverse. Many non-overlapping, up-regulated metabolic and cell-signaling pathways were identified in the first 3 time lapse comparisons. In contrast to the extensive changes in gene expression between 192 and 96 hours, fewer pathways were identified in the comparison between day 16 and day 8 (384 v 192h). The pathways regulating cell shape and structure as well as metabolic pathways were the few pathways that featured prominently during the 384 v 192 hr comparison. The data summarized here indicates that SGBS cells showed characteristic adipocyte features by 192 hours with subsequent transcriptional regulation targeted toward signaling networks that likely maintained the differentiated state. These data were consistent with our previous studies showing adipocyte specific metabolic flux was established at 192 hours but more robust at 384 hours^31^,^69^. A novel finding of these analyses was the up-regulation of the sodium independent glucose transporters between 384 and 192 hours. These transporters (*SLC2A5* and *SLC2A8*) are responsible for not only glucose uptake, but also fructose uptake^70^,^71^. A more detailed description of time lapse analysis is in the Supplementary file 1.

## Discussion

Analyzing mRNA abundance with microarrays and more recently RNA sequencing technologies is now a routine tool for high throughout analysis of cellular and tissue systems. However, the “data glut” makes it challenging for biologists to comprehensively interpret the results of sophisticated computational analysis, particularly when there is a very large number of differentially expressed genes. In many cases, the top 10 gene ontologies or the top 10 genes extracted from the high dimensional data are used to explain complex biological processes. Such approaches to data analysis limit the understanding of how cell or tissue systems operate.

Our novel network enrichment approach has advantages over other methodologies that use clustering and interacting genes or comparisons of submitted lists with annotated gene sets for functional analysis^2^,^72^,^73^. For example, clustering based on the gene ontology terms or expression profiles provides weak criteria for functional analysis and the predicted interactions between the gene products do not ensure that they are part of the same network – *i.e*., co-expression does not imply functional interaction. Another challenge of current enrichment analysis tools is their representation of results as list of GO terms and lack of condition specificity^72^. NASFinder reduces a list of molecules of interest after network mapping to a related group of molecules with a specific receptor/transporter, and improves discriminative power of gene sets for functional analysis. NASFinder improves the specificity of results by producing the tissue specific background network.

The networks and pathways identified by NASFinder confirmed and extend many independent studies of different pathways involved in adipogenesis which tend to analyze selected components of the system (e.g., transcription factor regulation during adipogenesis)^74^ or rely solely on p-values to rank discovered pathways. NASFinder provides a scoring system that ranks negatively and positively regulated pathways and networks and provides a score to indicate the activity of a given network in any comparison. Network activity scores show how different processes are occurring at a given time point (or between time points) but also how pathways are altered across time and differentiation. The results presented here can also be used to identify adipocyte systems processes within the newly described white adipose tissue systems network^75^.

We developed a (manual) process that maps pathways to a model cell. The position of these symbols represents the approximate cellular location of the majority of the components of the network since some members of a given cytoplasmic network (for example) may be localized to the cell membrane or nucleus (white lines in Figs 5 and 6 indicate this connection). These visualization tools allow for a more systematic view of how various pathways in the cell were regulated at a given time point or condition. An Excel file (Supplementary file 2-S2) consisting of a matrix of genes and identified pathways at each time point or time lapse was used to create Venn diagrams to show estimates of genes belonging to related pathways. A color code indicated up or down regulation for the pathway or network. For example, the 96 v 0 hr cell model (Fig. 5) shows the up-regulation of translational machinery, mitochondrial associated pathways, peroxisomal and PPAR signaling networks, and certain membrane associated complexes. The coordinate regulation of these pathways would be expected to play a role in cellular differentiation.

Global transcriptomic analysis and mapping of differentially expressed genes to networks uncovered another advantage of NASFinder. Gene families are often involved in complex cellular processes making it difficult to predict the biological roles of individual genes within the families. For example, membrane and extracellular matrix networks may be composed of different members of the integrin, laminin, and other membrane proteins interacting with different cellular structures (e.g., actin, also composed of different members of the family). The timing of gene expression may provide clues as to the function of a particular family member within the complex, assuming that – for example - timed transcriptional regulation during differentiation indirectly alters interactions of the protein complex through changes in protein levels. Although further biochemical studies are needed to prove any hypothesis generated from transcript abundance, the changes in expression may provide insights into how complex protein structures need to be regulated during the differentiation process. Since interactions of individual subunits with cognate partners of other complexes (e.g., integrins and actins) are often known, a more dynamic view of the regulatory and structural changes during differentiation may be elucidated.

Our analysis was limited to ‘receptors’ as the class of regulator molecules of interest, which may bias the results presented toward signaling pathways. However, metabolic and transcriptional regulatory sub-networks were also identified and often had high activity scores. Many of the networks identified with receptor as regulator molecules were also identified when using transcription factors and transporters as regulator molecules (not shown). We also used pathways for functional analysis instead of gene ontologies (which is an option in the NASFinder method) since our initial curation of the NASFinder output suggested that canonical pathways provided a more comprehensible explanation of the biological processes altered during adipogenesis. However, some of the pathway names relate to phenotype (*e.g.*, response to E. coli infection), which masks the underlying function (in this case, cytoskeleton) and necessitate further inspection of individual pathways.

NASFinder and the visualization tools described in this report provide valuable additions to the collection of tools for systems analysis of biological samples. Although this report focused on differentiating cells *in vitro*, no conceptual barrier exists to using these methods for other source material (*e.g.*, tissue biopsy) and for other types of molecules (*e.g.*, proteomics and metabolomics).

## Additional Information

**How to cite this article**: Nassiri, I. *et al*. Systems view of adipogenesis via novel omics-driven and tissue-specific activity scoring of network functional modules. *Sci. Rep.* **6**, 28851; doi: 10.1038/srep28851 (2016).

## Supplementary Material

Supplementary File 1

Supplementary File 2

Supplementary File 4

## Figures and Tables

**Table 1:** Number of Time Point Pathways by Functional Class.

TP Hr	Signaling Pathways	Transcription Factor	Metabolism	Energy	Membrane Structure	Cell Struct/Funct
6	43 (30)	–	4 (4)	–	–	–
48	56 (52)	4 (2)	12 (10)	3 (3)	12 (11)	14 (13)
96	74 (69)	9 (8)	25 (23)	7 (7)	17 (9)	12 (10)
192	66 (60)	6 (6)	32 (31)	9 (8)	11 (8)	11 (8)
384	74 (67)	8 (7)	27 (26)	4 (4)	17 (13)	5 (4)

Numbers in parenthesis are pathways with 50% or greater up-regulation within that class.

**Table 2:** Time Points Key Pathways (hr versus control).

Time point	Selected Processes	Result	Ref (e.g.)
6 v C	Decrease in cytokine signaling	Certain cytokines block adipogenesis	19,20,67
	Differential transcription factor regulation (e.g., *JUN, FOS*)	Cell cycle arrest, promotion of adipogenesis	76, 77, 78, 79
48 v C	Transamination	Decreased amino acid catabolism	68
	PPAR Signalling Pathway	Nuclear receptor signaling	80
	7 pathways associated with ribosome function	New protein synthesis	21
	Differential regulation of actin and related proteins	Changes in cytoskeletal structures	81, 82, 83
	Insulin	Insulin responsiveness	84
	Integrin	Cell surface remodelling	85
	Branched chain amino acid	Branched chain amino acid	68,86, 87, 88
	Synthesis of very long chain FA	Fat Metabolism	89
	Oxidative phosphorylation	Mitochondrial function	90
	Leptin	Leptin signalling	
96 v C	PPAR-γ pathway	PPAR signalling	74,91
	Focal adhesion/integrin	Cell remodelling	92
	Insulin signalling	Insulin responsiveness	84
	Pathogenic response to E. Coli	Tubulin and associated processes	82
	Leptin	Leptin signalling	67
	Pathways associated with ribosome function	New protein synthesis	21
192 v C	WNT signalling	Coupling signalling to transcription	93,94
	Insulin signalling	Insulin responsiveness	84
	Metabolism of proteins	Protein & mitochondrial synthesis	21
	PPAR, VDR_RXR	Nuclear receptor signalling	74,80,91
	Peroxisome	Fatty acid metabolism	
	IL5, ERK, ILK, P53	Cell signaling	
	Prolactin receptor signalling	Regulation of glucose/lipid & transporters	95
	Porphyrin metabolism	Linked to WNT signaling	96
	Pyruvate, Citrate and TCA cycle	Changes in energy metabolism	97
	Branched chain amino acid	Lipid & energy production	68,86,87
384 v C	Insulin pathway	Insulin responsiveness	84
	Glucose metabolism	Energy metabolism & precursors	67
	Adipokine networks	Cell signalling	98
	Integrin	Extracellular matrix	85
	Leptin	Adipokine signaling	67,98
	Cell cycle	Maintenance of cell state	99
	Branched chain amino acid	Lipid & energy production	68

**Table 3:** Number of Time Lapse Pathways by Functional Class.

TL	Signaling Pathways	Transcription Factor	Metabolism	Energy	Membrane Structure	Cell Struct/Funct
6 v 0	43 (30)	–	4 (4)	–	–	–
48 v 6	69 (63)	3 (3)	19 (17)	2 (2)	9 (5)	8 (5)
96 v 48	36 (35)	2 (2)	13 (12)	3 (3)	9 (2)	5 (3)
192 v 96	60 (49)	1 (1)	27 (26)	2 (2)	11 (1)	5 (1)
384 v 192	28 (28)	1 (1)	4 (2)	1 (1)	5 (5)	2 (2)

Numbers in parenthesis are pathways with 50% or greater up-regulation within that class.

**Table 4:** Time Lapse Key Pathways.

Time lapse	Selected Processes	Result	Ref (e.g.)
6 v 0	Decrease in cytokine signaling	Certain cytokines block adipogenesis	19,20,67
	Differential transcription factor regulation	Cell cycle arrest, promotion of adipogenesis	76, 77, 78, 79
48 v 6	IL signalling pathways	Signalling through JAK/STAT	67,100
	Peroxisome & UnsatFA synthesis	Fatty acid metabolism	101
	Prolactin receptor signalling	Regulation of glucose/lipid & transporters	95
	Pathogenic E. coli	Changes in cytoskeletal structures	81, 82, 83
	PPAR-γ pathway	Nuclear receptor signalling	74,80,91
	NOTCH2,3,4	Signalling	102,103
	TGF-β	Signalling	104
	Integrin	Extracellular matricx	85,105
96 v 48	PPAR-γ pathway	PPAR signalling	74,91
	Focal adhesion/integrin	Cell remodelling	92
	Insulin signalling	Insulin responsiveness	84
	Leptin & adipokine	Adipokine signaling	67,98
	Unsaturated fatty acid synthesis	Fatty acid metabolism	101
	Glyoxlate, dicarboxylate, glycine, serine, threonine	Precursors including for fatty acid biosynthesis	
	NOTCH, TGFB, TRIAL, CTCF	Cell signalling	102,103
	Citrate and TCA cycle	Changes in energy metabolism	97
	Syndecan	Link ECM to signalling pathways	106
	P53 pathway	Cell cycle control	107
192 v 96	WNT signalling	Coupling signalling to transcription	93,94
	Insulin signalling	Insulin responsiveness	84
	Glucose metabolism	Changes in energy metabolism	94
	PPAR signalling	PPAR signalling	74,91
	FOXM1	Multipl with DNA repair	108
	Peroxisome, FA metabolism	Fatty acid metabolism	101
	IL5, ERK, ILK, P53	Cell signaling	
	Porphyrin metabolism	Linked to WNT signaling	96
	Regulation of pyruvate dehydrogenase, Citrate & TCA cycle	Changes in energy metabolism	97
	Syndecan	Link ECM to signalling pathways	106
	Branched chain amino acid	Lipid biosynthesis	109
384 v 192	Na independent glucose transporters	Increased glucose & fructose uptake	
	FRA Pathway	Transcriptional regulations	98,110
	Integrin 4	Basement membrane structure	105
	NGF and Toll	Cell signaling	111
	IGF1 Pathway	Adipose regeneration	112
	Insulin signalling	Insulin responsiveness	84
	Cardiaegf	Calcium regulation	113
