# Supplementary material for: Systems view of adipogenesis via novel omics-driven and tissue-specific activity scoring of network functional modules
Source: Sci Rep. 2016 Jul 7;6:28851. doi: 10.1038/srep28851 (PMC4935943; doi:10.1038/srep28851)
Supplement: Supplementary File 1 [file srep28851-s1.pdf]

# Systems view of adipogenesis via novel omics-driven and tissue-specific activity scoring of network functional modules

Isar Nassiri[1], Rosario Lombardo[1], Mario Lauria[1], Melissa J. Morine[1], Petros Moyseos[1], Vijayalakshmi Varma[2], Greg T. Nolen[2], Bridgett Knox[2], Daniel Sloper[2], Jim Kaput[3*] and Corrado Priami[1,4,*]

# SUPPLEMENTARY FILE 1

Although the goal or our analysis was to provide a systems view of adipogenesis, much of the previous research in this field focused on either individual genes or subsets of genes in a pathway. To compare results obtained in our study with the published literature, we also provide below a more detailed analysis of data by pathway, gene, or sets of genes within a pathway. The lists of all pathways at a time point (TP) or between time points (Time Lapse, TL) are in Supplementary file 4 and the differentially expressed genes are reported in the TP and TL Crosstabs (Supplementary file 2-S2).

## 1. Histological staining of cells following initiation of adipogenesis

SGBS pre-adipocytes were induced to differentiate into adipocytes as described in the Material and Methods section of the main paper. A snapshot of cells at the different time points from the induction of differentiation is seen in the Supplement Fig. 1. The neutral lipid, Oil Red-O-stained cells display the characteristic phenotypical change with respect to lipid accumulation, as the pre-adipocyte differentiates into adipocytes.

### Oil Red O staining method

SGBS pre-adipocytes were cultured and induced to differentiate into adipocytes as described above. For Oil Red-O staining, at specific time points, the cells were gently washed twice with PBS and were fixed by exposing the cells to 10% formalin for 45 minutes, following which the cells were washed three times with deionized water. Fixed cells were stained with a commercially available

1 The Microsoft Research – University of Trento Centre for Computational and Systems Biology, Piazza Manifattura 1, 38068 Rovereto, Italy.
2 Division of Systems Biology, National Center for Toxicological Research, FDA, 3900 NCTR Road, Jefferson, AR 72079, USA
3 Nestlé Institute of Health Science, Lausanne, Switzerland.
4 Department of Mathematics, University of Trento, Via Sommarive 14, 38050 Povo, Italy.
I.N. and R.L. share first authorship. J.K. and C.P. share senior authorship. Communication to be addressed to C.P. (priami@cosbi.eu).

Oil Red O solution (Zen Bio Inc., Research Triangle Park, NC), that was filtered and then diluted with isopropanol in the ration 60:40 (Oil Red O solution to Isopropanol). The cells were stained with the diluted Oil Red O solution for 10 minutes following which the excess stain was washed using deionized water. Images of the stained cells were obtained using an AMG EVOS *xl* microscope (ThermoFisher Scientific Inc, Grand Island, NY).

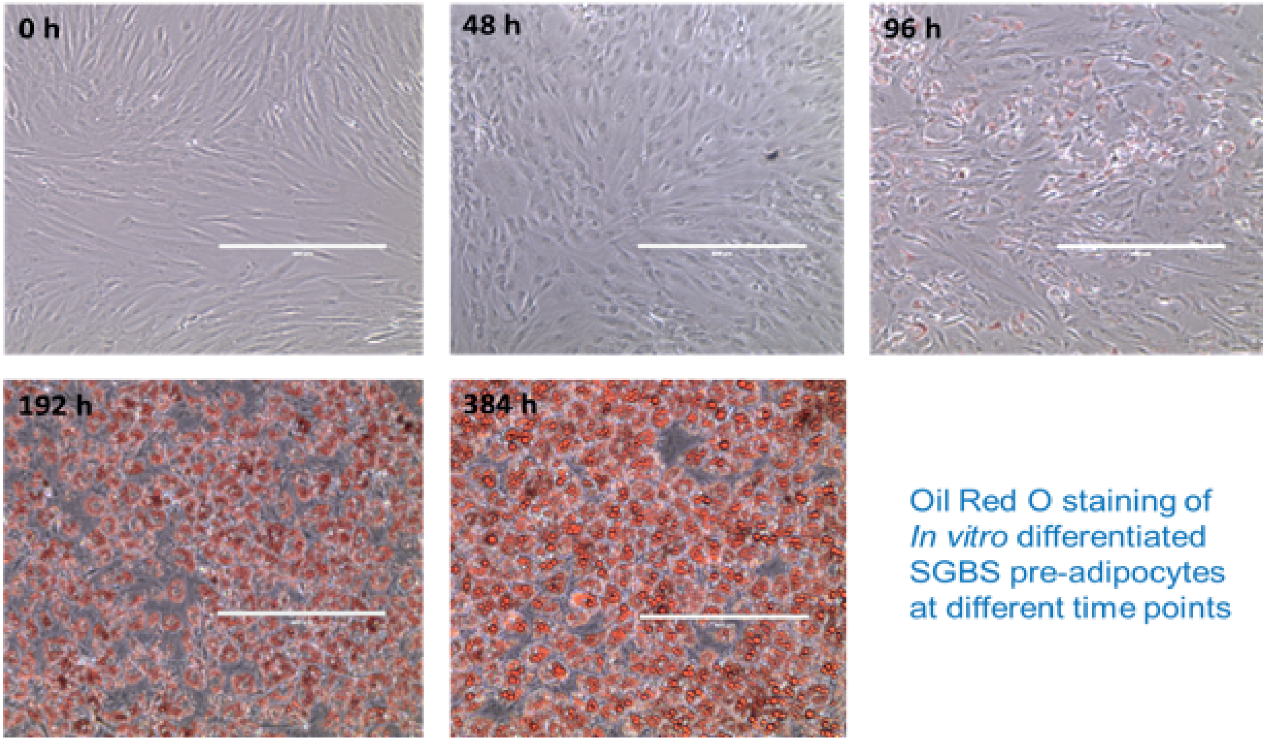


**Supplement Figure 1**: Oil Red-O staining of in vitro differentiated SGBS adipocytes. Differentiating adipocytes that were fixed using 4% PFA at 0, 48, 96, 192 and 384 hours from the induction of differentiation and were stained with Oil red O. Representative images of the Oil Red-O stained cells (red) at specific time points are depicted. Images were obtained using an AMG, EVOS xl microscope (ThermoFisher Scientific Inc, Grand Island, NY). Scale bars represent 400 µm.

## 2. NASFinder identified known pathways during adipogenesis: the Leptin Pathway receptor

The leptin transcriptional profile was analyzed across the differentiation process: a major function of mature adipocytes is the production of leptin[1, 2]. NASFinder identified changes in expression of genes in the leptin pathway at 48, 96, 192 and 384 hours (Supplement Fig. 2) after induction. Leptin receptor (LEPR) was the source node for this pathway and LEPR itself was differentially expressed over controls at 96 (1.4x), 192 (1.5x), and 384 (1.02x) hours but not at 0 or 6 hours. The common differentially expressed genes at 48, 96, 192, or 384 were *PRKAG1*, *PRKAG2*, *ACACA*,

*LEPR*, *PRKAA1*, and *CPT1A* (Supplement Table 1). The AMP kinase regulatory gene *PRKAG2* is up-regulated at each time point compared to progenitor cells reflecting the increased energy demands of the differentiation process and the role of this subunit in glucose metabolism[3, 4]. ACACA, the rate-limiting step in long chain fatty acid synthesis found by NASFinder in the network, is up-regulated during adipogenesis compared to its expression pre-differentiation (Supplement Table 3). Down-regulation of *CPT1A* is consistent with a change in energy metabolism from fatty acid oxidation of the progenitor cell to glucose.

**Supplement Table 1.**

**Main up-regulated and down-regulated genes of the Leptin pathway during adipogenesis.**

| Gene | Protein | Hours | | | |
|---|---|---|---|---|---|
| | | 48 | 96 | 192 | 384 |
| *PRKAG1* | AMP kinase noncatalytic gamma subunit 1 | 1.4 | 1.9 | 0.9 | 1.4 |
| *PRKAG2* | AMP kinase noncatalytic gamma subunit 2 | 1.5 | 2.1 | 3.2 | 2.2 |
| *ACACA* | Acyl-CoA carboxylase alpha | | 2.0 | 3.2 | 3.1 |
| *LEPR* | Leptin receptor | | 1.4 | 1.5 | 1.0 |
| *PRKAA1* | AMP kinase catalytic alpha subunit 1 | 1.0 | | 0.9 | |
| *CPT1A* | Carnitine palmitoyltransferase 1A | | -1.5 | -2.2 | -1.9 |

NASFinder demonstrated that many members of the network were found as nodes (*e.g.*, *TYK2, STAT5B, SOCS2, CALM3, CREBBP2*) throughout adipogenesis (48, 96, 192, 384 hrs). Additional 1-neighborhood networks indirectly connected to LEPR identified sub-networks which emerged transiently during differentiation. For example, genes involved in transcriptional regulation, *CREBBP2, NLRC4,* and *HIF1A*, were hubs for a large number of genes that may provide the foundation for changes in cell metabolism and structure that occur starting at 48 hours and continue through 192 hours (Supplementary file 3 - available at the URL http://www.cosbi.eu/3867/NASFinder_Supplementary_file_3.zip - tested only on Firefox).). Cells exhibited the prominent adipocyte feature of lipid accumulation (Supplement Fig. 1) and expression of key adipocyte genes including adiponectin, and leptin by 192 hours following the induction of differentiation as shown previously[5, 6]. DEGs connected to the nonDEG insulin receptor source node were transiently present at all time points relative to control.

Individual genes also demonstrated the dynamic changes in the leptin pathway. *TYK2*, one of 4 members of the Janus Kinase (JAK) family, was up-regulated (+1.1) at 48 hours, down-regulated (-1.1) at 96 hr, and a non-differentially expressed node versus control at 192 and 384 hrs. *TYK2*

connects the LEPR receptor to the *STAT3*, *STAT5A*, and *STAT5B* pathways that are involved in the transition from pre-adipocyte to mature adipocyte[6] (see below). Others have shown that the TYK2-STAT signaling pathway, which is regulated by phosphorylation events, are key processes in adipogenesis[7]. The transcriptomic analysis of the SGBS differentiation confirmed studies of individual genes connected to the leptin pathway and NASFinder placed these genes into the context of a topological protein interaction map.

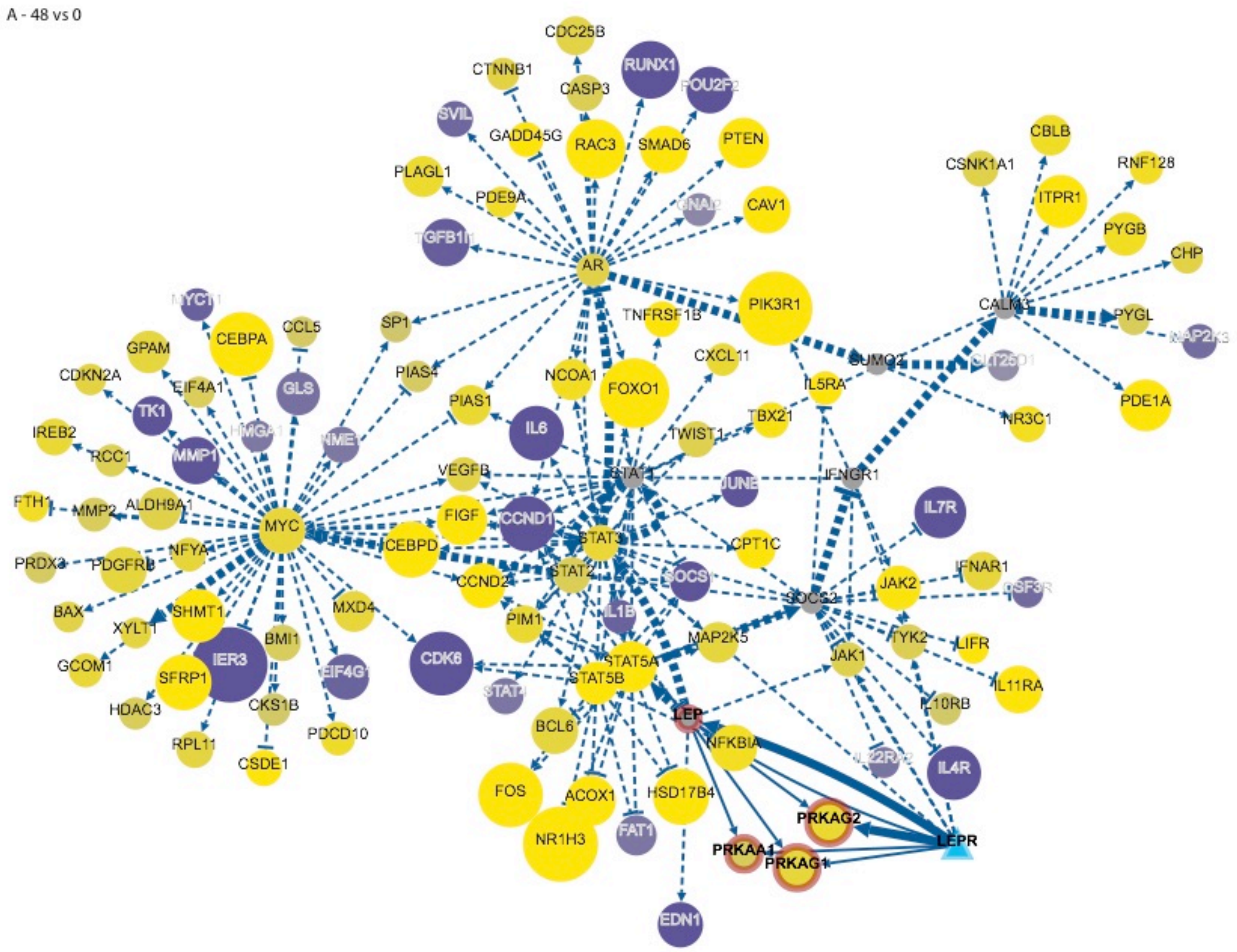

B - 96 vs 0

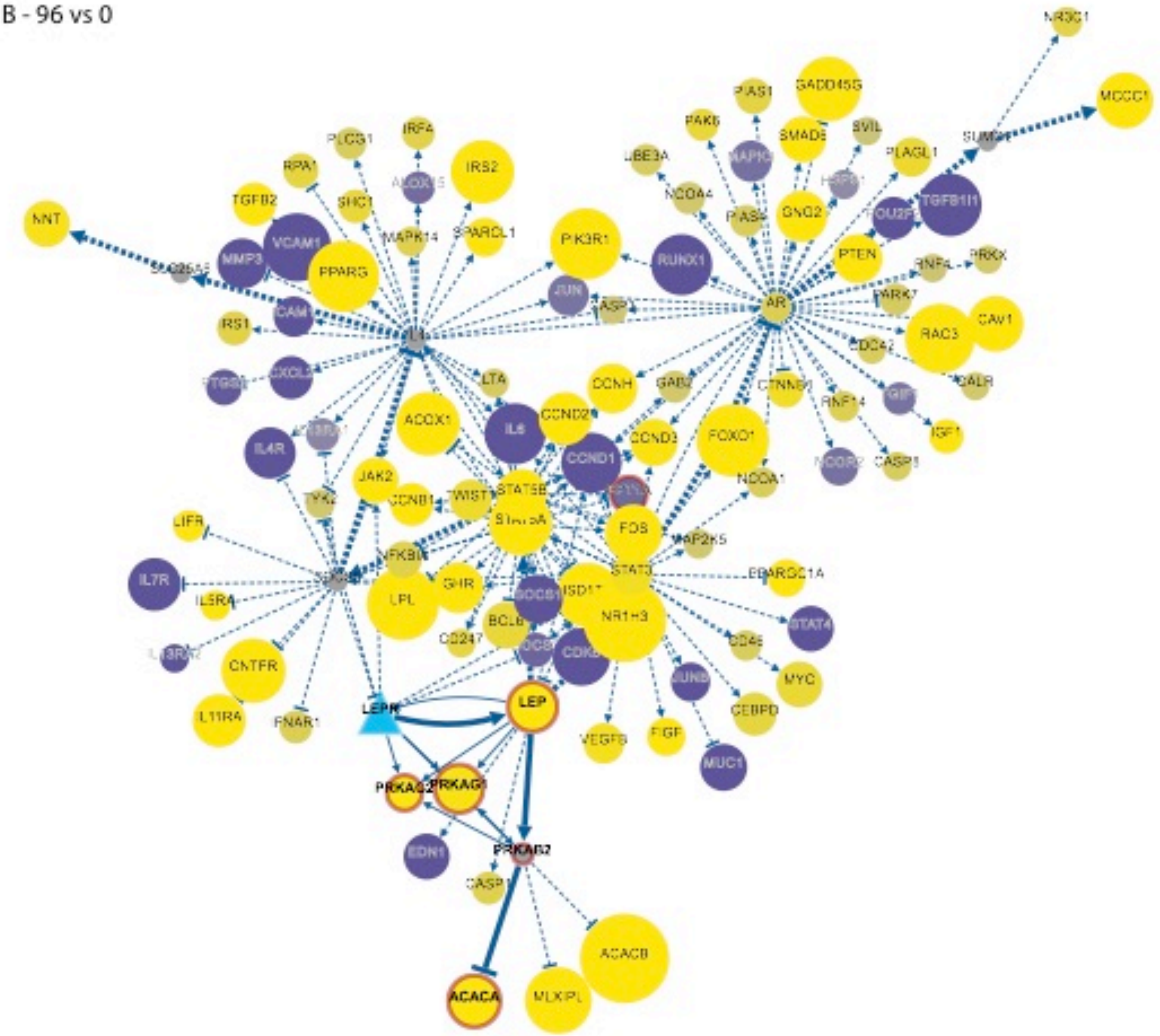
NR3C1
MCCC1
GADD45G
PIAS1
PAK6
SVIL
UBE3A
PLAGL1
NCOA4
PTEN
TGFB1I1
PRKX
AR
CAV1
RAC3
IGF1
CASP8
RNF14
IRF4
IRS2
PLCG1
RPA1
TGFB2
NNT
VCAM1
NMP3
PPARG
SHC1
MAPK14
SPARCL1
PIK3R1
RUNX1
JUN
LTA
CCNH
GAB2
ACOX1
IL6
CCND2
CCND1
CCND3
FOXO1
NCOA1
JAK2
IL4R
TYK2
LIFR
IL7R
IL5RA
CCNB1
TWIST1
STAT5B
FOS
MAP2K5
NFKBIA
LPL
GHR
BCL6
CD247
NR1H3
PPARGC1A
STAT4
JUNB
MYC
CEBPD
CNTFR
IL11RA
IFNAR1
LEPR
LEP
VEGFB
FIGF
MUC1
PRKAG2
PRKAG1
EDN1
PRKAB2
CASP1
ACACA
MLXIPL
ACACB

C - 192 vs 0

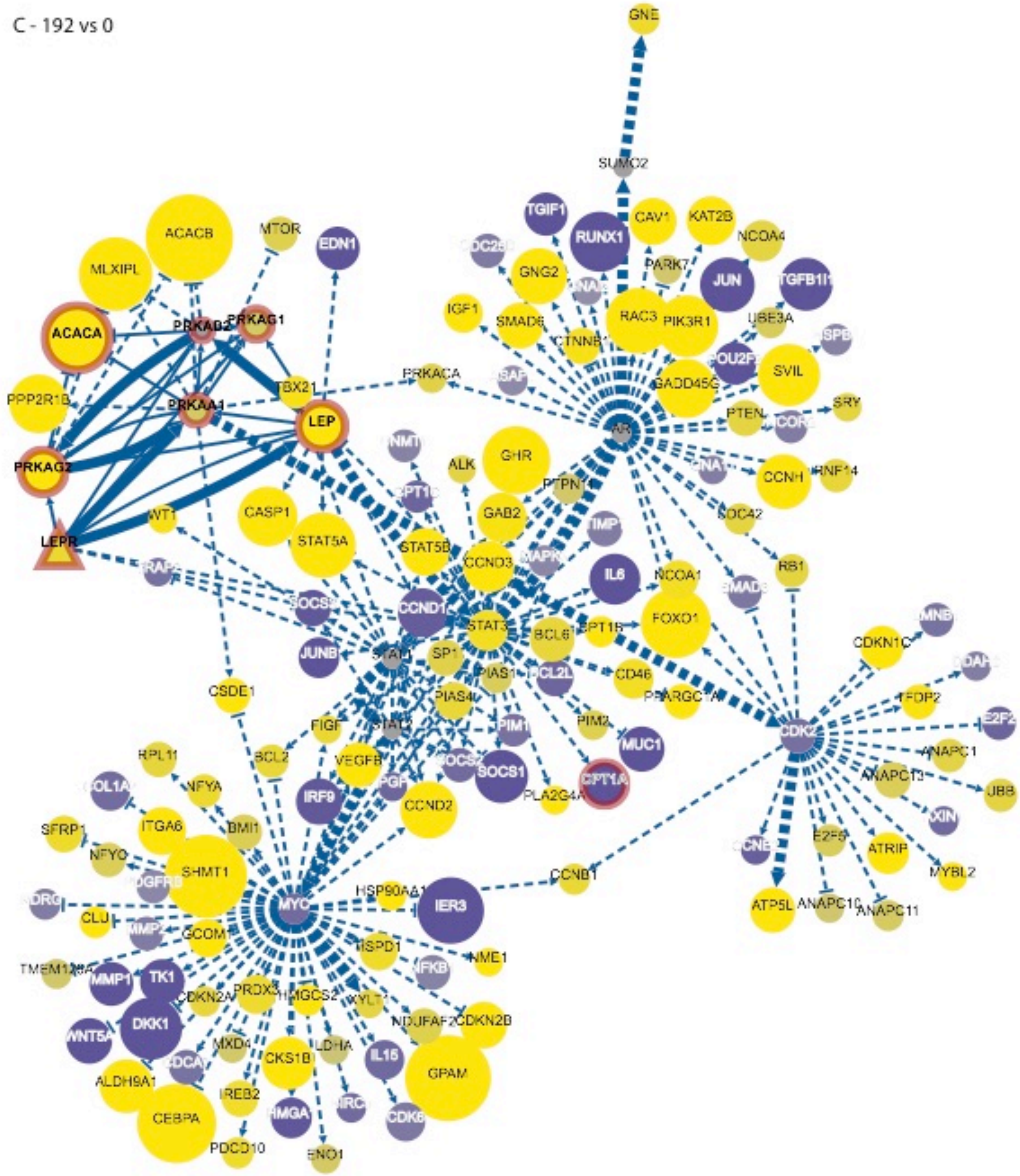

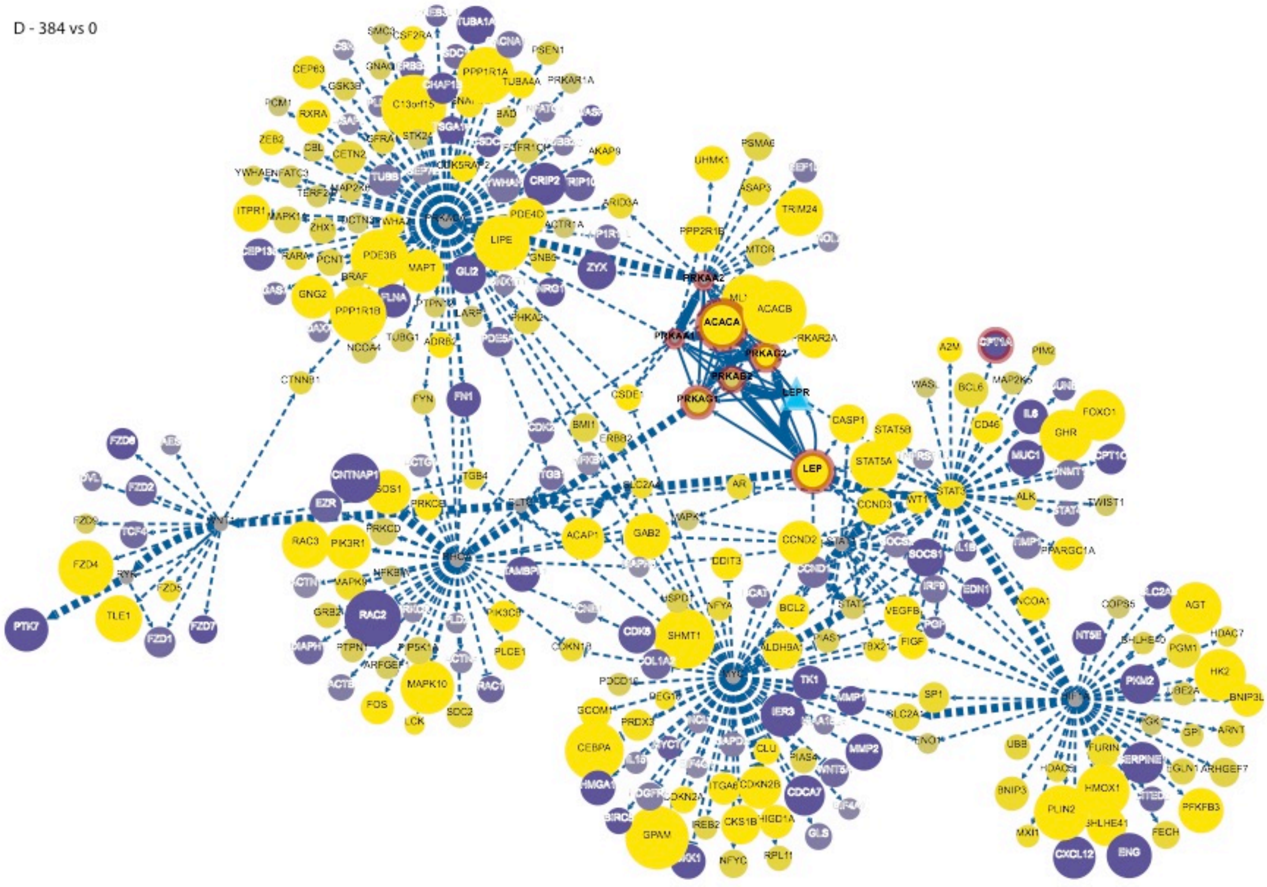


**Supplement Fig. 2.** The following figures A-D illustrate the changes in the Leptin pathway at each time point as seen by NASFinder. Each illustrated pathway is obtained as the intersection between two networks, the first being the subnetwork of the adipocyte interactome identified by NASFinder as active on the basis of the experimental evidence, and the second being canonical leptin pathway. Contextual experimental information is provided by the 1-neighborhood nodes (see Fig. 2 of main text for more details). The leptin receptor (LEPR) here is represented as a blue triangle.

## 3. Time Point Analysis

The specific transcriptomic changes occurring during the adipocyte differentiation process were interrogated using the NASFinder tool by time point (TP) and time lapse (TL) analytical strategies. The TP analysis, compared each time point against the control (0h time point), whereas in TL analysis each time point was compared with the previous one. All pathways discussed are statistically significant (p-value $\leq 0.05$)

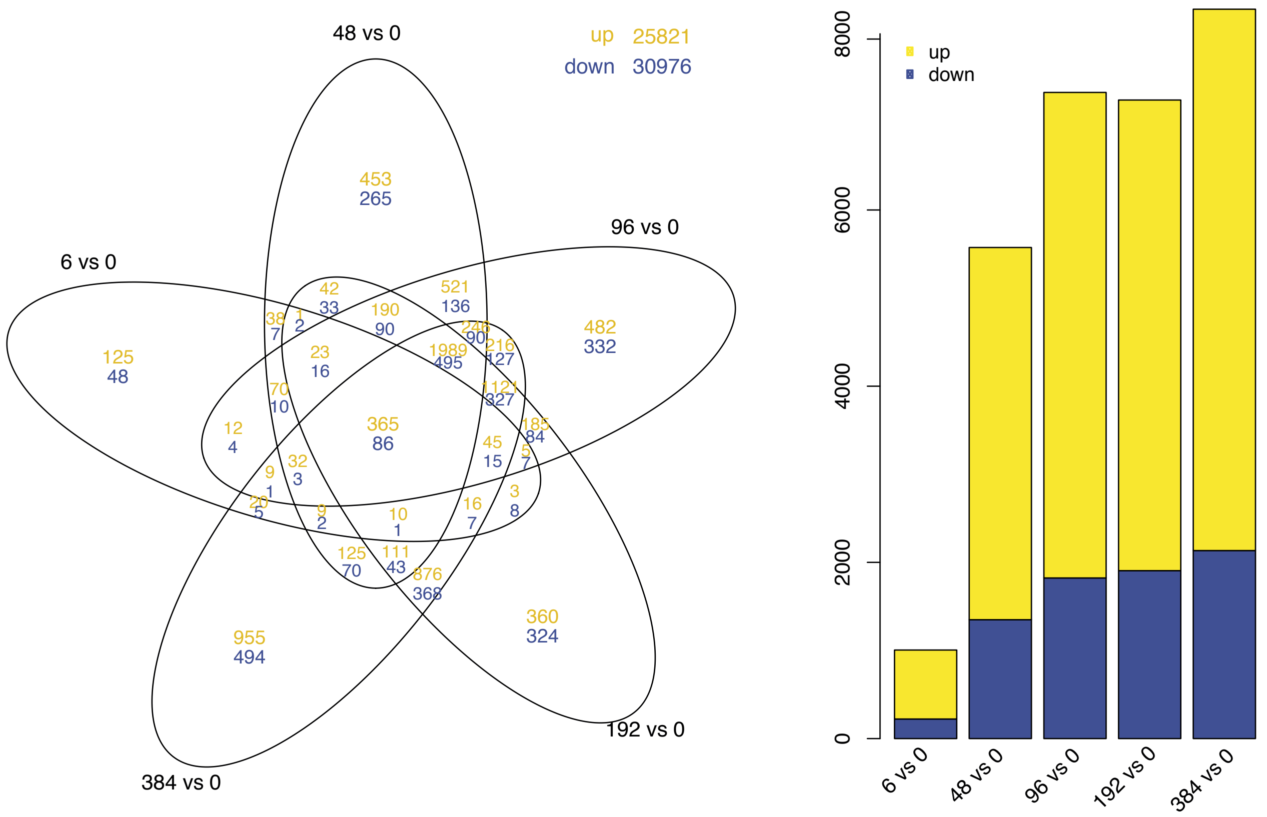


**Supplement Figure 3.** The Venn diagram and the histogram show the distribution of up/down regulated probes at each time point, showing how many responding probes are shared across different timepoints. In particular the probes at the center of the Venn diagram (365 up, and 86 down) are differentially expressed at each time point respect to 0h while the number of probes specific to each Venn set denote the differentially expressed probes specific to each time point.

## Supplement Table 2

### Fold Change of Genes Described for 6, 48, and 96 hr

| Genes @ 6 | Δ Fold | Protein |
|---|---|---|
| *ACSL1* | 2.9 | Acyl-CoA synthetase long-chain family member 1 |
| *AGTR1* | 1.5 | Angiotensin II receptor, type 1 |
| *ANGPTL4* | 3.1 | Angiopoietin-Like 4 |
| *eGFR* | 0.9 | epidermal growth factor receptor |
| *FOS* | 5.2 | FBJ Murine Osteosarcoma Viral Oncogene Homolog |
| *IL1A* | -2.3 | Interleukin 1, Alpha |
| *IL1B* | -2.7 | Interleukin 1, Beta |
| *IL6* | -4.9 | Interleukin 6, Beta |
| *IL12A* | -2.2 | Interleukin 12 |
| *IRAK3* | 2.3 | Interleukin 1 Receptor-Associated Kinase 3 |
| *JUN* | -1.6 | JUN Proto Oncogene |
| *PIK3R1* | 2.7 | Phosphoinositide-3-Kinase, Regulatory Subunit 1 (Alpha) |

| | | |
|---|---|---|
| *TSC22D3* | 4.8 | TSC22 Domain Family, Member 3 |
| **Genes @ 48** | **Δ Fold** | **Protein** |
| *ACTB* | -1.4 | Actin, Beta |
| *ARPC1B* | -1.9 | Actin related protein 2/3 complex, subunit 1B |
| *ARPC3* | -0.8 | Actin related protein 2/3 complex, subunit 3 |
| *ARPC5L* | -1 | Actin related protein 2/3 complex, subunit 5-like |
| *CAV1* | 3.2 | Caveolin 1 |
| *CCL2* | -2.3 | Chemokine (C-C Motif) Ligand 2 |
| *CSF1* | 2.4 | Colony Stimulating Factor 1 |
| *DCN* | 2.5 | Decorin |
| *FGR* | 1.2 | Feline Gardner-Rasheed Sarcoma Viral Oncogene Homolog |
| *FYN* | 2.2 | FYN Oncogene Related To SRC, FGR, YeS |
| *IL6* | -5.1 | Interleukin 6 |
| *JAK2* | 2 | Janus Kinase 2 |
| *KRT18* | -6 | Keratin 18 |
| *MGP* | 4.4 | Matrix gla protein |
| *PIK3R1* | 3.9 | Phosphoinositide-3-Kinase, Regulatory Subunit 1 (Alpha) |
| *PLAU* | -3 | Plasminogen Activator |
| *PLAUR* | -1.9 | Plasminogen Activator, Urokinase Receptor |
| **Genes @ 96** | **Δ Fold** | **Protein** |
| *ACSL1* | 4.6 | Acyl-CoA synthetase long chain |
| *CD36* | 7 | Fatty acid translocator protein |
| *NR1H3* | 6.2 | Liver X Nuclear Receptor Alpha Variant |
| *PPARG* | 3.3 | Peroxisome Proliferative Activated Receptor, Gamma |
| *RXRA* | 2.6 | Retinoid X Receptor, Beta |
| *RXRB* | -1.1 | Retinoid X Receptor, |
| *SCD* | 7.3 | Stearoyl CoA desaturase |
| *SCL27A1* | 1.8 | Fatty acid translocator protein |
| *TUBA1A* | -2.5 | Tubulin, Alpha |

***Time Point: 6 hrs versus Control***

Forty-nine sub-networks consisting of 210 genes (102 up-regulated) were identified by NASFinder with p-value < 0.05 at 6 hours post induction (Supplementary file 2-S2). Network activity scores (NAS) for pathways at 6 hours post-induction were generally lower (highest score was 0.21) compared to scores at other time points (up to NAS 0.43). The common feature of these pathways

at 6 hours was their involvement in cell signaling. Cytokine signaling sub-networks (IL27, IL1R, CD40) were generally down regulated (Supplement Table 2) at this time point and specifically, the key regulatory genes *IL1A, IL1B, IL6,* and *IL12A.* Down-regulation of these genes would decrease synthesis of extracellular cytokines that could block adipogenesis[8]. The 1 neighbor network of the AKAP13 pathway (NAS = 0.08, not shown) also contains one of the highest expressed genes in this dataset, *TSC22D3*, a leucine zipper transcriptional regulator involved in suppressing cytokine production. Down regulation of *JUN* and up-regulation of *FOS*, *PIK3R1*, *IRAK3* and *EGFR* are consistent with cell cycle arrest and promotion of differentiation processes[9-13]. Several anabolic pathways including synthesis of long chain fatty acids and purine metabolism were also induced at this time point.

***Time Point: 48 hours versus Controls***

One hundred and one [101] sub-networks were identified at 48 hours relative to control with 888 total and 645 up-regulated genes. A sub-network with one of the highest score was the FRA_Pathway (NAS = 0.29) with the source node RAPGEF5 (Rap guanine nucleotide exchange factor GEF 5). A subset of genes of this network encoded secreted proteins (e.g., *MMP1*, *PLAU*, *PLAUR*, *IL6*, *CCL2* – Supplement Table 2). Others have also documented changes in secreted protein expression throughout differentiation[14].

Eight of the overlapping networks involved protein synthesis including ribosome, peptide chain elongation, 3'-UTR translation regulation (NAS = 0.14), nonsense decay exon (NAS = 0.14), 43S complex formation (NAS = 0.16), mRNA metabolism (NAS = 0.11), MTOR (NAS = 0.04), and translational control (EIF4 pathway, NAS = 0.18), were differentially up-regulated at 48 hours relative to control. Two of these pathways were also up-regulated at 96 hours (MTOR, NAS = 0.26 and metabolism of mRNA, NAS = 0.16) along with the related transport of mature mRNA (NAS = 0.19) and spliceosome (NAS = 0.16). Dynamic changes in ribosome metabolism were previously characterized in 3T3-L1 cells and increased production of translational machinery is consistent with the need to produce adipocyte-specific proteins[15]. Actin and related proteins (*ACTB*, *ARPC1B, ARPC3*, *ARPC5L*, *KRT18*, and *TUBA1A*) were down-regulated which are indicative of alterations of the cytoskeleton (Supplement Table 2). Experimentally manipulated changes in the expression of the functionally related *Arp2/3* have been shown to alter adipogenesis of 3T3-L1 cells due to disruption of normal cytoskeletal remodeling[16].

Another "cluster" of pathways that shared between 2 and 5 genes was also induced at 48 hours relative to undifferentiated cells and included the insulin (NAS = 0.15), 5 overlapping integrin pathways (NAS = 0.17 to 0.22), and PTP1B (*PTPN1,* NAS = 0.16) networks. Overexpression of PTPN1 has been shown to inhibit adipogenesis in 3T3-L1 cells[17]. Other members of this network (e.g., *FYN, STAT5A*) were up-regulated (along with *STAT5B, PIK3R1, JAK2, FGR, TYK2, CAV1, CSF1* – Supplement Table 2), many of which are involved in processes that promote differentiation[13, 18, 19].

The circadian rhythm pathway (NAS = 0.3) was strongly induced at 48 hours while 50% of genes involved in the WNT signaling pathway were down-regulated[20]. *ARNTL* (+1.7 fold - arylhydrocarbon nuclear translocator protein, involved circadian rhythmicity) has been shown to suppress WNT signaling[21].

Other pathways identified at 48 hours, including those involved in central carbon metabolism (TCA cycle, NAS = 0.04), signaling (HEDGEHOG, NAS = 0.11; IL2RB NAS = 0.12, ARF (NAS = 0.19) and those in Figure 3 48 v 0), and transcriptional regulation (AP1, FOXM, FRA, HIF2), had medium (NAS = ~0.15) to high (NAS = > 0.2) NAS values and statistical significance. A full list of these pathways along with their statistics is in Supplementary file 2-S2.

***96 hours versus Control***

Based on work from our laboratory and others, 96 hours is about the half-way point to a fully differentiated adipocyte[22]. One hundred and forty-five sub-networks consisting of 1731 genes (1264 up-regulated) were differentially regulated at this time point. PPARγ, the central transcription factor in adipogenesis was up-regulated (3.2 fold) at 96 hours and was the source node for peroxisome (NAS = 0.25), metabolism of vitamins and cofactors (NAS = 0.26), and NFAT transcription factor pathway (NAS = 0.26). A subset of genes in these pathways is listed in Supplement Table 2. The NFAT pathway has been most studied in immune cells and cancer. This pathway integrates calcium signaling with transcriptional regulation[23] The transcription factors *RXRA*, *NR1H3*, and *RXRB* also were differentially regulated relative to pre-induction.

Two large networks had high NAS values at 96 hours: focal adhesion (NAS = 0.23) shared genes with the integrin, interleukin signaling, and ECM sub-networks (Fig. 3 in main paper and Supplementary file 2-S2). The integrin/focal adhesion networks were down-regulated (Supplement Table 3). However, the 96-hour "snap-shot" of 31 (of a total of 59 genes) up-regulated in the Focal

Adhesion network identified subsets of gene families that were oppositely regulated during this transition. For example, different members of the integrin, CD (cluster of differentiation), and laminin gene set were either up-regulated or down-regulated (Supplement Table 3) which may suggest that different combinations and interactions between these proteins alter complexes necessary for maintaining the shape of pre-adipocytes or the rounded morphology required for the adipogenesis process. Changes in cytoskeleton structure and its interactions with integral membrane proteins can be disrupted by mechanical stress or changes in the expression of key regulator genes such as calreticulin (rev in [24]).

The insulin signaling pathway (NAS = 0.21), which had 2 genes in common with mTOR, was also up-regulated at 96 hours. Two related pathways encoding cell structural components involving networks involving tubulin genes, post_chaperonin_tubulin folding (NAS = 0.22, down regulated) and pathogenic response to E. coli infection (NAS = 0.19) - involves microtubule destabilization, up-regulated) were also differentially regulated at 96 hours. Many of the other networks at 96 hours involved cell signaling processes (see Fig. 3a 96 vs 0 in main text and Supplementary file 2-S2).

**Supplement Table 3.**

**Focal Adhesion genes at the 96h timepoint (0 neighborhood).**

| Gene | **Δ Fold** | Protein |
|---|---|---|
| *FN1* | -1.5 | fibronectin 1 |
| *BCAR1* | -1.6 | breast cancer anti-estrogen resistance 1 |
| *RAC1* | -1.0 | ras-related C3 botulinum toxin substrate 1 |
| *CRK* | 1.2 | v-crk sarcoma virus CT10 oncogene homolog (avian) |
| *ITGB5* | -1.0 | integrin, beta 5 |
| *ITGA3* | -3.5 | integrin, alpha 3 (antigen CD49C, alpha 3 subunit of VLA-3 receptor) |
| *ITGB4* | 1.9 | integrin, beta 4 |
| *ITGB7* | 1.4 | integrin, beta 7 |
| *SHC1* | 1.5 | SHC (Src homology 2 domain containing) transforming protein 1 |
| *SOS1* | 2.5 | son of sevenless homolog 1 (Drosophila) |
| *COL1A1* | -0.9 | collagen, type I, alpha 1 |
| *ITGA10* | -1.8 | integrin, alpha 10 |
| *ITGA5* | -2.1 | integrin, alpha 5 (fibronectin receptor, alpha polypeptide) |
| *LAMA1* | -3.0 | laminin, alpha 1 |
| *LAMA2* | 4.7 | laminin, alpha 2 |
| *LAMA5* | -2.3 | laminin, alpha 5 |
| *LAMB1* | 1.9 | laminin, beta 1 |
| *LAMB2* | -1.7 | laminin, beta 2 (laminin S) |
| *PDGFRB* | 0.8 | platelet-derived growth factor receptor, beta polypeptide |

| | | |
|---|---|---|
| *JUN* | -1.3 | jun oncogene |
| *PIK3R1* | 3.8 | phosphoinositide-3-kinase, regulatory subunit 1 (alpha) |
| *MAPK3* | -1.4 | hypothetical LOC100271831; mitogen-activated protein kinase 3 |
| *PTEN* | 2.1 | phosphatase and tensin homolog |
| *IGF1* | 3.7 | insulin-like growth factor 1 (somatomedin C) |
| *IGF1R* | 1.1 | insulin-like growth factor 1 receptor |
| *RAF1* | -0.7 | v-raf-1 murine leukemia viral oncogene homolog 1 |
| *CAV1* | 3.1 | caveolin 1, caveolae protein, 22kDa |
| *ILK* | -0.7 | integrin-linked kinase |
| *EGFR* | -0.7 | epidermal growth factor receptor (ev-erb-b) |
| *MAPK10* | 4.8 | mitogen-activated protein kinase 10 |
| *MAPK9* | 0.9 | mitogen-activated protein kinase 9 |
| *CCND1* | -2.4 | cyclin D1 |
| *GSK3B* | 1.0 | glycogen synthase kinase 3 beta |
| *PAK1* | -1.0 | p21 protein (Cdc42/Rac)-activated kinase 1 |
| *ACTN1* | -1.5 | actinin, alpha 1 |
| *CTNNB1* | 1.7 | catenin (cadherin-associated protein), beta 1, 88kDa |
| *VCL* | -1.9 | vinculin |
| *ZYX* | -1.1 | zyxin |
| *CAPN2* | -0.9 | calpain 2, (m/II) large subunit |
| *ACTB* | -1.0 | actin, beta |
| *ACTN4* | -1.1 | actinin, alpha 4 |
| *CAV2* | 2.4 | caveolin 2 |
| *CCND2* | 3.0 | cyclin D2 |
| *CCND3* | 1.9 | cyclin D3 |
| *CDC42* | 1.0 | cell division cycle 42 (GTP binding protein, 25kDa) |
| *FYN* | 1.5 | FYN oncogene related to SRC, FGR, YES |
| *LAMA4* | 1.3 | laminin, alpha 4 |
| *MYL12A* | -2.0 | myosin, light chain 12A, regulatory, non-sarcomeric |
| *MYL5* | 2.3 | myosin, light chain 5, regulatory |
| *MYL9* | -2.6 | myosin, light chain 9, regulatory |
| *PARVA* | 0.7 | parvin, alpha |
| *PDGFD* | 1.5 | platelet derived growth factor D |
| *PPP1CB* | 0.8 | protein phosphatase 1, catalytic subunit, beta isoform; speedy homolog |
| *PPP1CC* | 1.1 | protein phosphatase 1, catalytic subunit, gamma isoform |
| *RAC2* | -2.3 | rho family, small GTP binding protein Rac2 |
| *RAC3* | 4.3 | ras-related C3 (rho family, small GTP binding protein Rac3) |
| *RAPGEF1* | 1.0 | Rap guanine nucleotide exchange factor (GEF) 1 |
| *RELN* | -3.3 | reelin |
| *TLN2* | 2.5 | talin 2 |

***192 hours versus Controls***

The total number of differentially expressed genes increased slightly between 96 (1731 genes) hours and 192 hours (1902) and the percentage of up-regulated genes was very similar (73 and 75%, respectively). One hundred and thirty-seven networks were identified by NASFinder at 8

days after induction. The highest scoring pathways were regulation of the signaling pathway IGF activity (NAS = 0.42), energy metabolism (Citrate TCA cycle, NAS = 038 and valine, isoleucine, and leucine degradation, NAS = 0.4), and several metabolic and biosynthetic pathways (fatty acid metabolism, triglyceride biosynthesis, proponate, pyruvate, glyoxylate and dicarboxylate metabolism, all between NAS 0.38 and 0.35). Others have shown that branched chain amino acid (BCAA) degradation promoted lipid biosynthesis, that this pathway was expressed during differentiation branched chain amino acid (BCAA) degradation has been shown to promote lipid biosynthesis, and was sensitive to levels of leucine and inflammatory status in 3T3-L1 cells[25, 26]. Many of these pathways are considered as defining the mature adipocyte.

The cell cycle pathway (NAS = 0.17) was noticeable because of the number of genes [92] involved, 67% of which were up-regulated. (Supplement Table 4). Major components of this pathway include several anaphase promoting complex/cyclosome (APC/C) proteins as well as members of the proteasome (PSM) protein family (see Supplement Table 4). Interpreting how individual proteins contribute to the structure and regulatory properties of APC/C is challenging because of the structural complexity and interactions with components of diverse cellular processes[27]. Comparing the transcriptional regulation at 192 and 384 hours post induction (Supplement Table 4) may allow for development of hypotheses for different subunits based on the cellular state at each time point. Stable isotope mapping demonstrated differences in metabolic flux between these time points suggesting (perhaps) that pathways involved in cell cycle regulation were not completely established at 192 hours – correlating changes in expression of individual genes at each time point may help identify those involved in maintaining the fully differentiated adipocyte (Supplement Table 4)[22].

**Supplement Table 4.**

**Cell Cycle Proteins at 192 and 384 Hour**

| Gene | Δ Fold @ 192h | Δ Fold @ 384h | Protein |
|---|---|---|---|
| ACTR1A | 1.0 | 0.8 | ARP1 actin-related protein 1 homolog A, centractin alpha |
| AHCTF1 | | 1.3 | AT hook containing transcription factor 1 |
| AKAP9 | 2.6 | 2.1 | A kinase (PRKA) anchor protein (yotiao) 9 |
| ANAPC1 | 1.3 | 0.9 | anaphase promoting complex subunit 1 |
| ANAPC10 | 0.8 | 1.0 | anaphase promoting complex subunit 10 |
| ANAPC11 | 1.0 | 1.1 | anaphase promoting complex subunit 11 |
| APITD1 | | 0.8 | cortistatin; apoptosis-inducing, TAF9-like domain 1 |

| | | | |
|---|---|---|---|
| ATRIP | 2.0 | 2.0 | ATR interacting protein |
| AURKB | | -2.7 | aurora kinase B |
| BIRC5 | -1.5 | -2.1 | baculoviral IAP repeat-containing 5 |
| BUB3 | -0.6 | | budding uninhibited by benzimidazoles 3 homolog (yeast) |
| CCDC99 | | -2.1 | coiled-coil domain containing 99 |
| CCNB1 | 1.5 | | cyclin B1 |
| CCNB2 | | -1.9 | cyclin B2 |
| CCND1 | -1.8 | -1.4 | cyclin D1 |
| CCND2 | 3.6 | 3.9 | cyclin D2 |
| CCND3 | 2.3 | 2.0 | cyclin D3 |
| CCNE1 | | -1.0 | cyclin E1 |
| CCNE2 | -1.8 | | cyclin E2 |
| CCNH | 2.8 | 2.2 | cyclin H |
| CDC20 | | -2.3 | cell division cycle 20 homolog (S. cerevisiae) |
| CDC25B | -1.2 | | cell division cycle 25 homolog B (S. pombe) |
| CDK2 | -1.4 | | cyclin-dependent kinase 2 |
| CDK6 | -1.2 | | cyclin-dependent kinase 6 |
| CDC7 | | -1.7 | cell division cycle 7 homolog (S. cerevisiae) |
| CDK1 | | -1.5 | cell division cycle 2, G1 to S and G2 to M |
| CDK2 | | -1.5 | cyclin-dependent kinase 2 |
| CDK5RAP2 | | 2.0 | CDK5 regulatory subunit associated protein 2 |
| CDK6 | | -1.9 | cyclin-dependent kinase 6 |
| CDKN1B | | 1.6 | cyclin-dependent kinase inhibitor 1B (p27, Kip1) |
| CDKN2A | 1.4 | 2.0 | cyclin-dependent kinase inhibitor 2A (melanoma, p16, inhibits CDK4) |
| CDKN2B | 2.1 | 3.1 | cyclin-dependent kinase inhibitor 2B (p15, inhibits CDK4) |
| CDKN2C | 8.2 | 8.3 | cyclin-dependent kinase inhibitor 2C (p18, inhibits CDK4) |
| CENPL | 1.1 | | centromere protein L |
| CENPN | 0.9 | | centromere protein N |
| CEP135 | -1.2 | -1.8 | centrosomal protein 135kDa |
| CEP63 | 1.9 | 1.8 | centrosomal protein 63kDa |
| CEP72 | | -0.9 | centrosomal protein 72kDa |
| CETN2 | 1.6 | 1.4 | centrin, EF-hand protein, 2 |
| CHEK1 | | -1.0 | CHK1 checkpoint homolog (S. pombe) |
| CHEK2 | -1.8 | -1.3 | protein kinaseCHK2 checkpoint homolog (S. pombe |
| CKS1B | 2.2 | 2.2 | CDC28 protein kinase regulatory subunit 1B |
| CLASP1 | 0.8 | | cytoplasmic linker associated protein 1 |
| CLIP1 | | 1.3 | CAP-GLY domain containing linker protein 1 |
| CSNK1D | -0.9 | -0.9 | casein kinase 1, delta |
| CSNK1E | -1.0 | -1.0 | casein kinase 1, epsilon |
| CUL1 | 1.1 | 0.9 | cullin 1 |
| DCTN3 | | 0.9 | dynactin 3 (p22) |
| DNA2 | 2.3 | | DNA replication helicase 2, yeast homolog |
| E2F2 | -1.6 | -1.6 | E2F transcription factor 2 |
| E2F5 | 1.0 | 1.0 | E2F transcription factor 5, p130-binding |
| ERCC6L | -1.8 | | excision repair cross-complementing rodent repair deficiency |
| FBXO5 | -1.5 | -1.1 | F-box protein 5 |
| FGFR1OP | 0.9 | 1.1 | FGFR1 oncogene partner |
| H2AFX | -1.2 | | H2A histone family, member X |

| | | | |
|---|---|---|---|
| HDAC1 | 0.9 | | histone deacetylase 1 |
| HIST2H2AC | | 3.2 | histone cluster 2, H2ac |
| HSP90AA1 | 2.9 | | heat shock protein 90kDa alpha (cytosolic), class A member2 |
| HSPA2 | 2.6 | 2.0 | heat shock 70kDa protein 2 |
| KIF20A | -2.1 | -2.0 | kinesin family member 20A |
| KIF2A | | 1.0 | kinesin heavy chain member 2A |
| LMNB1 | | -1.7 | lamin B1 |
| MAD1L1 | | -1.3 | MAD1 mitotic arrest deficient-like 1 (yeast) |
| MAX | 1.3 | | MYC associated factor X |
| MCM10 | -2.1 | -2.4 | minichromosome maintenance complex component 10 |
| MCM2 | | -1.9 | minichromosome maintenance complex component 2 |
| MCM3 | -0.6 | -0.9 | minichromosome maintenance complex component 3 |
| MCM4 | | -2.0 | minichromosome maintenance complex component 4 |
| MCM5 | | -1.3 | minichromosome maintenance complex component 5 |
| MCM6 | | -0.9 | minichromosome maintenance complex component 6 |
| MCM7 | | -1.2 | minichromosome maintenance complex component 7 |
| MYBL2 | 2.0 | | v-myb myeloblastosis viral oncogene homolog (avian)-like 2 |
| MYC | -1.5 | | v-myc myelocytomatosis viral oncogene homolog (avian) |
| NDEL1 | -0.7 | | nudE nuclear distribution gene E homolog (A. nidulans)- 1 |
| NUF2 | | -1.2 | NUF2, NDC80 kinetochore complex component, homolog |
| ORC2 | | -0.9 | ORC2 |
| PCM1 | | 0.7 | pericentriolar material 1 |
| PCNT | 1.2 | 1.3 | pericentrin |
| PKMYT1 | -1.6 | -2.6 | protein kinase, membrane associated tyrosine/threonine 1 |
| PLK4 | | -1.2 | polo-like kinase 4 (Drosophila) |
| POLD1 | -1.2 | -1.3 | polymerase (DNA directed), delta 1, catalytic subunit 125kDa |
| POLD2 | 1.9 | 1.3 | polymerase (DNA directed), delta 2, regulatory sub 50kDa |
| POLD3 | 1.7 | | polymerase (DNA-directed), delta 3, accessory subunit |
| PPP2R1B | 6.4 | 4.9 | protein phosphatase 2, regulatory subunit A, beta isoform |
| PPP2R5A | 3.9 | 3.9 | protein phosphatase 2, regulatory subunit B', alpha isoform |
| PPP2R5C | 1.2 | 1.2 | protein phosphatase 2, regulatory subunit B', gamma |
| PRKACA | 1.3 | | protein kinase, cAMP-dependent, catalytic, alpha |
| PSMA1 | 2.1 | 1.7 | proteasome (prosome, macropain) subunit, alpha type, 1 |
| PSMA2 | 1.5 | | proteasome (prosome, macropain) subunit, alpha type, 2 |
| PSMA4 | 1.2 | 1.1 | proteasome (prosome, macropain) subunit, alpha type, 4 |
| PSMA6 | 1.3 | 1.3 | proteasome (prosome, macropain) subunit, alpha type, 6 |
| PSMB1 | 1.1 | 0.9 | proteasome (prosome, macropain) subunit, beta type, 1 |
| PSMB10 | 1.4 | 0.8 | proteasome (prosome, macropain) subunit, beta type, 10 |
| PSMB3 | 0.7 | | proteasome (prosome, macropain) subunit, beta type, 3 |
| PSMB5 | 1.5 | 1.3 | proteasome (prosome, macropain) subunit, beta type, 5 |
| PSMB8 | -2.0 | -1.6 | proteasome (prosome, macropain) subunit, beta type, 8 |
| PSMB9 | -2.8 | -1.9 | proteasome (prosome, macropain) subunit, beta type, 9 |
| PSMC1 | 1.0 | 0.8 | proteasome (prosome, macropain) 26S subunit, ATPase, 1 |
| PSMC4 | 1.2 | 1.3 | Proteasome (prosome, macropain) 26S subunit, ATPase, 4 |
| PSMC6 | 1.2 | 1.1 | proteasome (prosome, macropain) 26S subunit, ATPase, 6 |
| PSMD1 | 0.8 | | proteasome (prosome, macropain) 26S subunit, non-ATPase, 1 |
| PSMD10 | 1.8 | 2.1 | proteasome (prosome, macropain) 26S subunit, non-ATPase, 10 |
| PSMD11 | 1.5 | 0.9 | proteasome (prosome, macropain) 26S subunit, non-ATPase, 11 |
| PSMD12 | 1.0 | | proteasome (prosome, macropain) 26S subunit, non-ATPase, 12 |

| | | | |
|---|---|---|---|
| PSMD13 | 1.3 | 1.1 | proteasome (prosome, macropain) 26S subunit, non-ATPase, 13 |
| PSMD4 | 1.1 | 1.1 | proteasome (prosome, macropain) 26S subunit, non-ATPase, 4 |
| PSMD8 | 2.5 | 2.6 | proteasome (prosome, macropain) 26S subunit, non-ATPase, 8 |
| PSME1 | -1.0 | -0.9 | proteasome (prosome, macropain) activator subunit 1 (PA28 a) |
| PSMF1 | 0.8 | 0.9 | proteasome (prosome, macropain) inhibitor subunit 1 (PI31) |
| PTTG1 | | -1.6 | pituitary tumor-transforming 1 |
| RAD17 | | 1.7 | RAD17 homolog (S. pombe) |
| RAD21 | | 1.9 | RAD21 homolog (S. pombe) |
| RAD9A | | 1.7 | RAD9 homolog A (S. pombe) |
| RB1 | 1.4 | 1.4 | retinoblastoma 1 |
| RBBP4 | 1.3 | 1.5 | hypothetical LOC642954; retinoblastoma binding protein 4 |
| RBL2 | | 1.6 | retinoblastoma-like 2 (p130) |
| RPA1 | 1.3 | 1.4 | replication protein A1, 70kDa |
| RPA2 | 0.9 | 1.0 | replication protein A2, 32kDa |
| RPS27 | -1.1 | -0.9 | ribosomal protein S27 |
| RRM2 | | -2.5 | ribonucleotide reductase M2 polypeptide |
| RUVBL2 | 1.2 | | RuvB-like 2 (E. coli) |
| SKP2 | | -1.2 | S-phase kinase-associated protein 2 (p45) |
| SMC3 | | 0.8 | structural maintenance of chromosomes 3 |
| STAG2 | | 1.0 | stromal antigen 2 |
| SUN2 | 1.2 | | unc-84 homolog B (C. elegans) |
| TERF2 | 1.0 | 1.0 | telomeric repeat binding factor 2 |
| TERF2IP | | 1.0 | telomeric repeat binding factor 2, interacting protein |
| TUBA1A | -2.3 | -2.7 | tubulin, alpha 1a |
| TUBA4A | | 2.7 | tubulin, alpha 4a |
| TUBB | -1.2 | -1.4 | tubulin, beta |
| TUBG1 | 1.3 | 1.0 | tubulin, gamma 1; similar to Tubulin, gamma 1 |
| TYMS | -1.4 | -2.0 | thymidylate synthetase |
| UBE2C | -2.9 | -3.6 | ubiquitin-conjugating enzyme E2C |
| UBE2E1 | | 1.5 | ubiquitin-conjugating enzyme E2E 1 (UBC4/5 homolog, yeast) |
| YWHAE | | 1.2 | Mitochondrial import stimulation factor L subunit) (MSF L) |

The PPAR signaling (NAS = 0.33) and peroxisome (NAS = 0.29) were also up-regulated at 192 hours, as expected for this central adipogenic pathway. NASFinder also identified other transcription factor (e.g., AR, NAS = 0.22) and cell signaling pathways (e.g., IL5, NAS = 0.3; ERK, NAS = 0.2; ILK, NAS = 0.26; and P53, NAS = 00.2) that were not as well studied from the better characterized adipocyte-specific networks.

***384 hours versus Controls***

Maintenance of the differentiated state required up-regulation of 1140 of 1619 (~70%), which encompassed 137 networks identified by NASFinder. Many of these networks shared genes resulting in clustering of transcriptional, structural, signaling, and metabolic pathways. The insulin pathway (NAS = 0.25) was connected through co-expression of 2 to 8 genes with many other signaling pathways (e.g., IGF1, NAS = 0.22; PTP1B, NAS = 0.21; ERBB1, NAS = 0.18, and

other). The cell cycle pathways (NAS = 0.19; Supplement Table 4) share cyclin and checkpoint genes (down-regulated *CCND1*, *CDK1, CDK2, CCNB2*, *CHEK2*, and up-regulated *CDKN2A* and *CKS1B*) with the FOXM1 network (NAS = 0.3) connecting transcriptional regulation with maintenance of the non-proliferative mature adipocyte. The VDR_RXR (NAS = 0.27), PPARA (NAS = 0.28) and nuclear receptor transcription factor networks (NAS = 0.15), and *PPARG* gene (+3.9 fold) also remained up-regulated.

Several glucose transporters were also up-regulated at 384 hours relative to control: *SLC2A1* (+1.5 – *GLUT1*), and *SLC2A4* (+1.7 – *GLUT4*) while *SLC2A3* (-1.9 – *GLUT3*) was down-regulated. *GLUT4* is the insulin responsive glucose transporter and *GLUT3* normally functions in fetal tissues[28]. Other central metabolic processes including propanoate, TCA cycle, valine, leucine, and isoleucine (BCAA) biosynthesis, and transport of vitamins were also induced at 384 hours over controls and had similar regulation to the 192 time point. The change of BCAA degradation at 192 hours to biosynthesis at 384 hours may help explain the increased release of BCAA observed in conditions of insulin resistance[29]. A number of other signaling pathways were also altered relative to the pre-adipocyte (see Supplementary file 2-S2).

The establishment of the adipocyte-specific transcriptional pattern for each network involved the contribution of many genes, some of which transiently expressed. *MAPK3* was modestly down-regulated at 96 and 192 hours and up-regulated at 384. *MAPK3* is a GWAS locus for T2DM, and is among the genes at the intersection of adipose- and muscle-transcriptome and TGF-β signaling pathway in a calculated T2DM interactome[30]. Phosphatidylinositol 3-kinase regulatory subunit 1 (*PIK3R1*) was differentially expressed between all time points and pre-differentiation and was involved in over 20% of all pathways at each time point (Supplement Table 5). Transcription of PI3KR1 is done by PPARγ[18] and, in turn, this regulatory subunit helps orchestrate not only adipogenesis but also the mature adipocyte state through many related and unrelated pathways (see TP crosstab file).

**Supplement Table 5**

**PIK3R1 Regulation & Pathways**

| TP (Hr) | PIK3R1 Level | Total Pathways | Pathways with PIK3R1 | % of total with PIK3R1 |
|---|---|---|---|---|
| 6 | 2.7 | 49 | 17 | 34.7 |
| 48 | 3.9 | 102 | 33 | 32.4 |
| 96 | 3.8 | 145 | 35 | 24.1 |
| 192 | 3.1 | 137 | 29 | 21.2 |
| 384 | 3.0 | 138 | 38 | 27.5 |

## 4. Time Lapse Analysis

We compared fold changes at each time point to those at time zero, and additionally we compared fold changes between pairs of consecutive time points, considering all possible pairs (6 versus 0, 48 versus 6, 96 versus 48, etc). NASFinder comparison of (for example) 48 versus 6 hour may (and does) differ from 48 hour versus 0 hour. Expression of genes that differ between sequential time points elucidates active processes contributing to adipocyte formation. The following Supplement Fig. 4 summarizes the number of significantly up/down regulated time probes for the five time lapses (*right*), and the amount of overlap between the five lists (*left*).

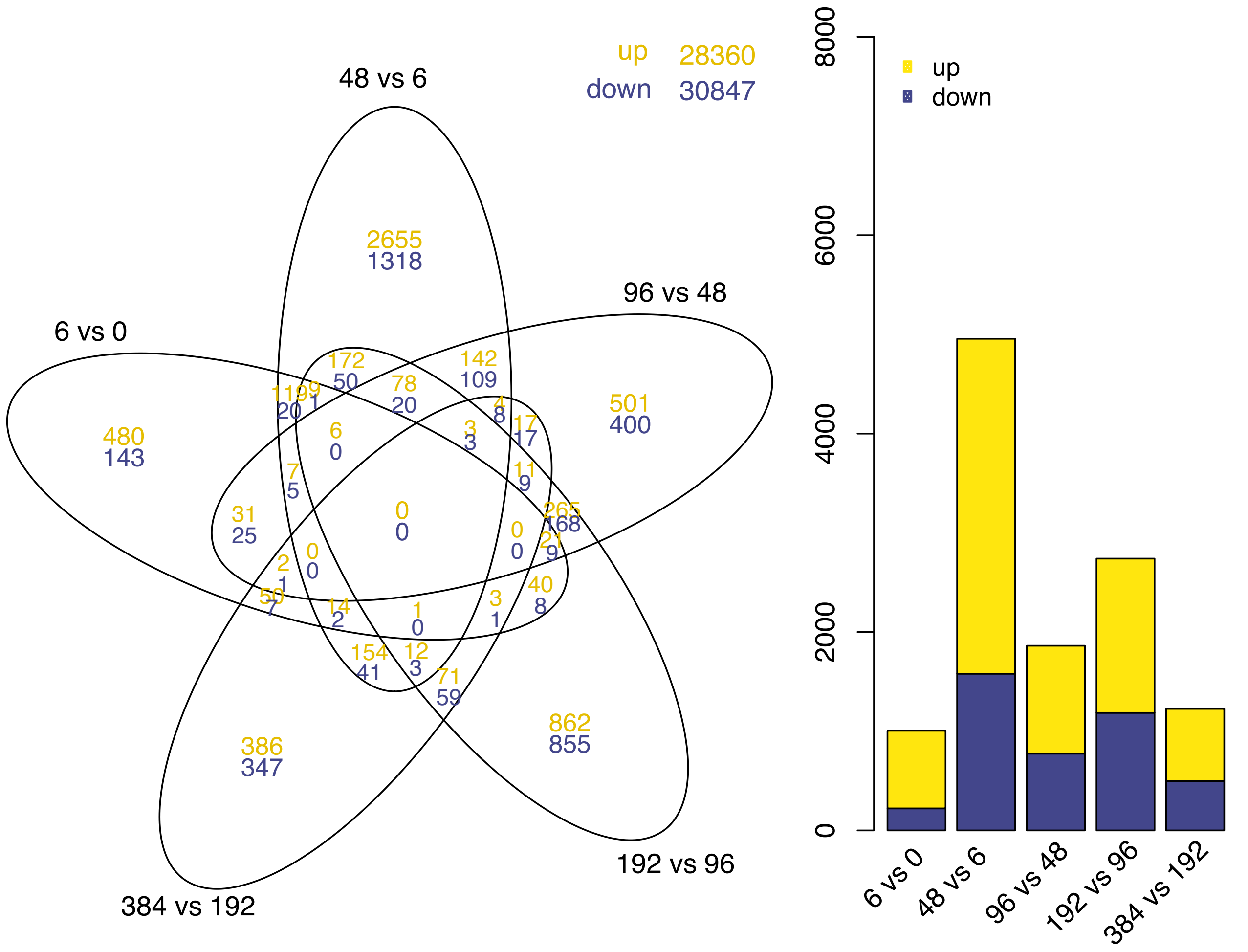


**Supplement Figure 4**: the distribution of up/down regulated probes at each lapse shows where most probes are significantly different from previous time point. **File FigS_4.**

***Time Lapse: 48 versus 6 hours***

NAS Finder identified 112 consolidated pathways that differed between 6 hours and 48 hours consisting of 779 genes of which were 493 were up-regulated (63%). Pathways related to cytoskeleton (response to E. coli pathogen – NAS = 0.26), insulin (NAS = 0.14), insulin receptor recycling NAS = 0.17), and multiple signaling pathways:  adipocytokine (NAS = 0.13), IGF growth factor (NAS = 0.32).  Metabolic pathways involving glutathione (NAS = 0.2), pentose (NAS = 0.18), glycolysis and gluconeogenesis (NAS = 0.18), branched chain amino acid degradation (NAS = 0.27), and DNA repair (NAS = 0.11) were up-regulated while amino acid synthesis (NAS = 0.28), and vitamins and cofactors (NAS = 0.18) were down-regulated at 48 hours compared to 6 hours.

Two integrin pathway (Integrin4 [NAS = 0.28] and A9B1 [NAS = 0.22]) were up-regulated while Integrin 3 (NAS = 0.18) and A6B1_A6B4 (NAS = 0.22) were down-regulated demonstrating the

selectivity of membrane structures that change during conversion of pre-adipocytes to adipocytes. The source node receptor for integrin 4 pathway (100% up-regulated), *ITGA4* (integrin alpha 4), has not previously been described in adipocytes but is known to be associated with the ITGA6 in cells such as epithelial cells, Schwann cells etc[31]. ITGA6 was demonstrated to support the clustering of growth arrested preadipocytes on the basement membrane, a critical step in establishing contact inhibition and preventing the preadipocytes from re-entering the cell cycle[32]. The integrin 3 pathway was mediated via the TGFBR2 and TGFBR3 receptors which were 66.7 and 100 % down regulated, respectively, at 48 hours compared to the 6 hour time point. These pathways were marginally up-regulated in mouse 3T3L1 adipocytes[33]. While many of the pathways identified by NASFinder were independent of each other at the 0-neighborhood level, a number of sub-networks shared genes indicating coordinate regulation of these complexes (Fig. 3 and 4 in main paper). Transcriptomic results could provide the molecular detail of how various protein complexes participate in the forming the extracellular and intracellular processes for differentiation to occur with the caveat that transcription is not equivalent to protein levels[34].

Additional networks that are altered related to cell structure include the Notch signaling-2 (NAS = 0.18) and Notch3 (NAS = 0.18) pathways, which were both down regulated by 50% relative to the 6 hour time point. The role of Notch signaling is still to be fully defined across adipogenesis (e.g., [35] versus [36]). The sub-network proteolysis of P75NTR (NAS = 0.16) occurs through the presenilin-dependent-secretase that regulates TRK receptors and Notch signaling[37].

The hallmark pathways of adipogenesis, the PPAR network (NAS = 0.22), and sub-networks (PPAR signaling and nuclear receptor signaling) and the related RXR-VDR (NAS = 0.19) and downstream peroxisome pathways (NAS = 0.18) were up-regulated between 48 hours and 6 hours. Proinflammatory signaling pathways (IL2 PI3K, IL2RB, IL2, IL4, NAS range of 0.13 to 0.15), were down regulated between 6 and 48 hours leading to decreased cytokine signaling and promotion of adipogenesis. However, the anti-inflammatory pathways including IL4 (NAS = 0.27) and IL5 (NAS = 0.24) mediated pathways were up-regulated supporting the adipogenic process. While anti-inflammatory cytokines including IL1RA and IL13 (which were not DEG) have been shown to be augmented at earlier stages of differentiation (day 7 compared to days 14 and 21)[38], the specific early (48h) changes in IL-4 and IL-5 anti-inflammatory pathways during adipogenesis have not been previously described. The TGF-β receptor signaling pathway (NAS = 0.15) was up-regulated consistent with results that show TGF-β inhibits adipogenesis[39].

A notable difference observed when comparing 48 hours versus 6 hours (time lapse) and 48 hours and 0 (time point) is the absences of sub-networks involved in translational machinery, indicating that these pathways were continuously expressed across this time period.

### *Time Lapse: 96 versus 48 hours*

Differential gene expression decreased between 96 hours and 48 hours with 238 of 416 genes being up-regulated (57%) across 70 pathways (Fig. 3 in main paper). The up-regulated leptin pathway had the highest NAS (0.28) during this period (Fig. 3 and 4 in main paper and leptin section). The focal adhesion (NAS = 0.08), integrin1 (NAS = 0.17), integrin3 (NAS = 0.14), and urokinase-type plasminogen activator (NAS = 0.18) sub-networks were down-regulated in the time lapse comparison, similarly to the time point analysis between 96 and 0 hours. The genes involved in these pathways (Supplement Table 6) overlap and connect to cell signaling or are regulated by genes involved in transcription. These transcriptional changes are consistent with membrane restructuring that occurs during differentiation[34].

**Supplement Table 6**

**Differentially Expressed Genes involved in Cell Membrane Function**

| Gene | Δ Fold | FOCAL_ADHESION | ECM_RECEPTOR_INTERACTION | INTEGRIN_CS | INTEGRIN1 | INTEGRIN3 | SYNDECAN_4 | UPA_UPAR | INTEGRIN_CS_INTERACTIONS | Protein |
|---|---|---|---|---|---|---|---|---|---|---|
| *CAV2* | 1.4 | 1 | | | | | | | | caveolin 2 |
| *CCND3* | 1.3 | 1 | | | | | | | | cyclin D3 |
| *COL5A1* | -0.6 | 1 | 1 | | | | | | | collagen, type V, alpha 1 |
| *EGFR* | 0.0 | 1 | | | | | | | | epidermal growth factor receptor l (v-erb-b) |
| *FN1* | -1.7 | 1 | 1 | | 1 | 1 | 1 | 1 | 1 | fibronectin 1 |
| *FYN* | -0.8 | 1 | | | | | | | | FYN oncogene related to SRC, FGR, YES |
| *ITGA3* | -0.8 | 1 | 1 | 1 | 1 | | | 1 | 1 | integrin, alpha 3 (antigen CD49C) |
| *ITGA5* | -1.4 | 1 | 1 | | 1 | | 1 | 1 | 1 | integrin, alpha 5 (fibronectin receptor, α |
| *ITGA6* | 2.9 | 1 | 1 | 1 | 1 | | | | | integrin, alpha 6 |
| *ITGA7* | 1.8 | 1 | 1 | | 1 | | | | | integrin, alpha 7 |
| *ITGB5* | -0.9 | 1 | 1 | 1 | | | | 1 | 1 | integrin, beta 5 |

| | | | | | | | | | | |
|---|---|---|---|---|---|---|---|---|---|---|
| *JUN* | -1.4 | 1 | | | | | | | | jun oncogene |
| *LAMA5* | -2.1 | 1 | 1 | | 1 | | | | 1 | laminin, alpha 5 |
| *MAPK3* | -0.7 | 1 | | | | | | | | hypothetical LOC100271831; mitogen-activated protein kinase 3 |
| *PDGFC* | 1.2 | 1 | | | | | | | | platelet derived growth factor C |
| *PDGFRA* | -0.9 | 1 | | | | | | | | platelet-derived growth factor receptor, α |
| *PDGFRB* | -0.6 | 1 | | | | 1 | | 1 | | platelet-derived growth factor receptor, beta |
| *PLAU* | -1.1 | | | | 1 | 1 | | | | plasminogen activator, urokinase |
| *PLAUR* | -1.0 | | | | 1 | | | 1 | | plasminogen activator, urokinase receptor |
| *RAC2* | -1.1 | 1 | | | | | | | | ras-related C3 botulinum toxin substrate 2 |
| *RAC3* | 1.3 | 1 | | | | | | | | ras-related C3 botulinum toxin substrate 3 |
| *SHC1* | 1.2 | 1 | | | | | | | 1 | SHC (Src homology) transforming protein 1 |
| *TGFBI* | -1.1 | | | | 1 | | | | | transforming growth factor, beta-induced |
| *THBS1* | -1.0 | 1 | 1 | | 1 | 1 | 1 | | 1 | thrombospondin 1 |
| *TNC* | -1.4 | 1 | 1 | | 1 | | 1 | | | tenascin C |
| *ZYX* | -0.8 | 1 | | | | | | | | zyxin |

Four Notch signaling pathways were up-regulated between 96 and 48 hours: Notch2, Notch3, Notch4 had NAS scores of 0.22 and activated Notch1 with NAS of 0.13. These pathways share *PSEN2* (+2.7 fold), *JAG1* (+1.3), *JAG2* (+1.3) and *DL1* (-1.2), the last three being ligands for the activation of Notch signaling in the nucleus pathway. Others have shown that blocking Notch signaling with inhibitors of γ-secretase blocks adipogenesis[40].

PPAR (NAS = 0.17) and PPARA (NAS = 0.13) signaling pathways remained induced between 96 and 48 hours reflecting the importance of this transcriptional network in programming differentiation. Pathways downstream of these transcriptional networks were also up-regulated (e.g., Peroxisome with NAS = 0.09).

Increased abundance of genes involved in several metabolic pathways including biosynthesis of triglyceride (NAS = 0.22), fatty acid (NAS = 0.15) and unsaturated fatty acids (NAS = 0.22), Metabolism of glycine, serine, and threonine (NAS = 0.11) and sulfur amino acids (NAS = 0.1) were also up-regulated across this interval. Cysteine is pro-adipogenic[41] and is a precursor of taurine. The taurine pathway was induced during differentiation of 3T3-L1 cells and rat adipose tissue[42], but this pathway was found only at 48hr v 0 comparisons in this study. Amino acid sensing has been proposed to play a key role in adipogenesis and couples changes in amino acid levels with decreased maintenance of germ stem cells in Drosophila[43]. The role of metabolism of amino acid changes in adipogenesis remains unclear.

***Time Lapse: 192 versus 96 hours***

Time lapse analysis identified 618 genes differentially expressed between 192 and 96 hours with 245 up-regulated (56%). NASFinder identified 106 pathways containing these genes. Although signaling pathways were the predominant class, 27 metabolic pathways were found with all but the histidine metabolism pathway (NAS = 0.1) being up-regulated. High scoring metabolic networks with commonly DEGs (Supplement Table 7) included fatty acid metabolism (NAS = 0.19), fatty acid triacylglycerol metabolism (NAS = 0.15), and degradation valine, isoleucine, and isoleucine degradation (NAS = 0.22), all of which were up-regulated. Branched chain amino acids may provide energy for fatty acid biosynthesis[44]. Several overlapping integrin and related pathways were also up-regulated between 192 and 96 hr including integrin 1 (NAS = 0.24), integrin A9B1 (NAS = 0.13), and syndecan 4 (NAS = 0.22) while extracelluar matrix degradation pathways (NAS = 0.15) were 100% down-regulated or 50% down-regulated (CS_DS_Degradation, NAS = 0.33). These results imply that remodeling of the cell membrane may be nearing completion at 192 hours. The majority (75%) of the intracellular signaling pathways had NAS scores below NAS = 0.15 suggesting decreased importance in the differentiation process (see Supplementary file 2-S2).

***Time Lapse: 384 versus 192 hours***

In contrast to the extensive changes in gene expression during other time lapse periods, only 131 genes were differentially expressed between 384 and 192 hours with 83 up-regulated. These genes mapped to 39 pathways with only one pathway score above 0.2 (Na+ independent glucose transporters NAS = 0.23). Most (31/39 = 79%) of the pathways had NAS below 0.1 suggesting that these pathways were involved in final maturation and maintenance of the adipocyte phenotype established at 8 days. The CardiacEGF (NAS = 0.13), IGF1 (NAS = 0.11), NGF (NAS = 0.10), CDK5 (NAS = 0.09) and 7 other pathways were linked by their transcription factors *FOS* (+2.2), *JUN* (+1.6) or both. The PPAR pathways were not differentially expressed between these time points suggesting that the JUN/FOS network regulated signaling pathways essential for mature adipocytes[13]. Son of sevenless (SOS, +1.1) was involved in 11 pathways. *SOS1* plays key roles in growth regulation through the RAS pathway and in regulating the structure of the cytoskeleton through interactions with actin[45] but has not been well studied in adipogenesis or the mature adipocyte.

## Supplement Table 7

## Genes Involved in Fatty Acid Metabolims at 192 versus 96 hours

| *Gene* | Δ Fold | FATTY_ACID_METABOLISM | PPAR_SIGNALING_PATHWAY | VALINE_LEUCINE_AND_ISOLEUCINE_DEGRADATION | FATTY_ACID_TRIACYLGLYCEROL & KETONE_BODY_METABOLISM | MITOCHONDRIAL_FATTY_ACID_BETA_OXIDATION | TRIGLYCERIDE_BIOSYNTHESIS | Protein |
|---|---|---|---|---|---|---|---|---|
| *ACSL3* | 1.2 | 1 | 1 | | 1 | | 1 | acyl-CoA synthetase long-chain family 3 |
| *ACADM* | 1.9 | 1 | 1 | 1 | 1 | 1 | | acyl-Coenzyme A dehydrogenase, C-4 to C-12 |
| *ECHS1* | 1.5 | 1 | | 1 | 1 | 1 | | enoyl Coenzyme A hydratase, 1, mitochondrial |
| *HADH* | 0.9 | 1 | | 1 | 1 | 1 | | hydroxyacyl-Coenzyme A dehydrogenase |
| *HADHA* | 0.9 | 1 | | 1 | 1 | 1 | | hydroxyacyl-Coenzyme A dehydrogenase/3-ketoacyl-Coenzyme A thiolase/enoyl-Coenzyme A hydratase, alpha subunit |
| *HADHB* | 0.7 | 1 | | 1 | 1 | 1 | | hydroxyacyl-Coenzyme A dehydrogenase/3-ketoacyl-Coenzyme A thiolase/enoyl-Coenzyme A hydratase, beta subunit |
| *ACADVL* | 1.0 | 1 | | | 1 | 1 | | acyl-Coenzyme A dehydrogenase, very long chain |
| *ECI1* | 0.9 | 1 | | | 1 | 1 | | ECI1 |
| *ACAT1* | 0.7 | 1 | | 1 | 1 | | | acetyl-Coenzyme A acetyltransferase 1 |
| *ACAA1* | 1.1 | 1 | 1 | 1 | | | | acetyl-Coenzyme A acyltransferase 1 |
| *ACAT2* | 1.8 | 1 | | 1 | | | | acetyl-Coenzyme A acetyltransferase 2 |
| *ALDH3A2* | 1.5 | 1 | | 1 | | | | aldehyde dehydrogenase 3 family, member A2 |
| *CPT1B* | 3.3 | 1 | 1 | | 1 | | | choline kinase β; carnitine palmitoyltransferase 1B |
| *CPT2* | 1.5 | 1 | 1 | | 1 | | | carnitine palmitoyltransferase 2 |

| | | | | | | | | |
|---|---|---|---|---|---|---|---|---|
| *CPT1C* | -2.1 | 1 | 1 | | | | | carnitine palmitoyltransferase 1C |
| *ACAA2* | 1.6 | 1 | | | | | | acetyl-Coenzyme A acyltransferase 2 |
| *GK* | 1.4 | | 1 | | 1 | | 1 | glycerol kinase 3 pseudogene; glycerol kinase |
| *GPAM* | 1.8 | | | | 1 | | 1 | glycerol-3-phosphate acyltransferase, mitochondrial |
| *LPIN1* | 0.7 | | | | 1 | | 1 | lipin 1 |
| *HMGCS1* | 0.8 | | | 1 | 1 | | | 3-hydroxy-3-methylglutaryl-Coenzyme A synth 1 |
| *MVD* | 3.6 | | | | | | | mevalonate (diphospho) decarboxylase |
| *FDFT1* | 0.9 | | | | 1 | | | farnesyl-diphosphate farnesyltransferase 1 |
| *PCCB* | 1.1 | | | 1 | 1 | | | propionyl Coenzyme A carboxylase, beta |
| *MUT* | 1.0 | | | 1 | 1 | | | methylmalonyl Coenzyme A mutase |
| *BCKDHB* | 1.6 | | | 1 | | | | branched chain keto acid dehydrogenase E1, beta |
| *HSD17B10* | 0.9 | | | 1 | | | | hydroxysteroid (17-beta) dehydrogenase 10 |
| *IVD* | 1.2 | | | 1 | | | | isovaleryl Coenzyme A dehydrogenase |
| *MCCC1* | 1.0 | | | 1 | | | | methylcrotonoyl-Coenzyme A carboxylase 1 α |

## *Additional Figures and Tables*

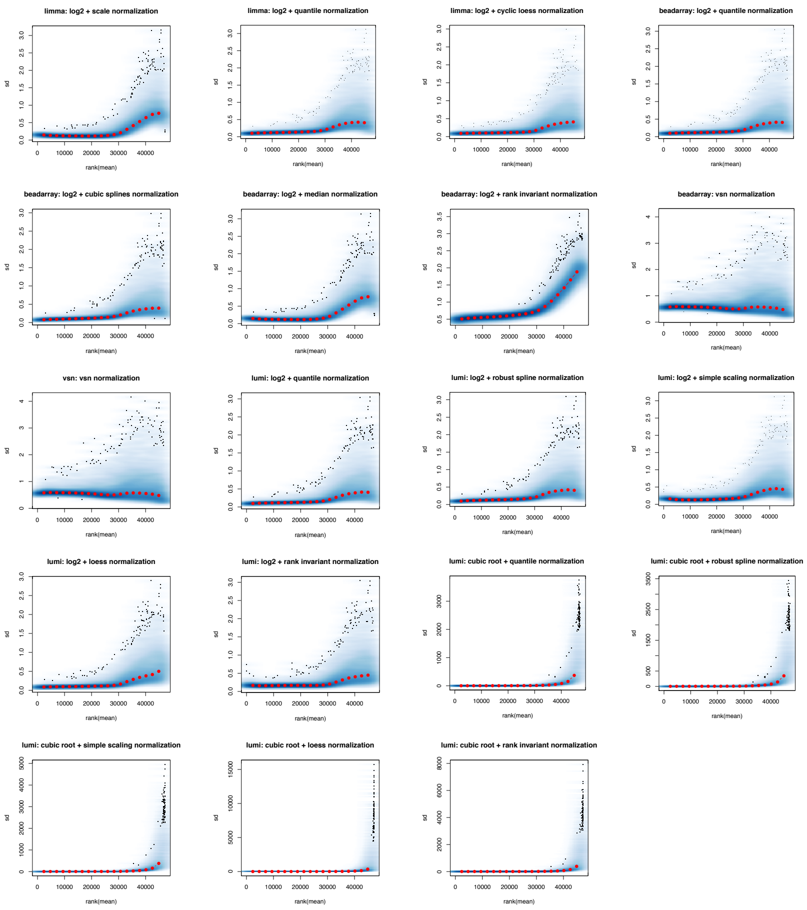


**Supplement Figure 5.** The plots of the empirical standard deviation versus the rank of the mean computed for the list of 19 variance stabilization / normalization methods tested. The plots were obtained using the diagnostic functions in the VSN package[26].

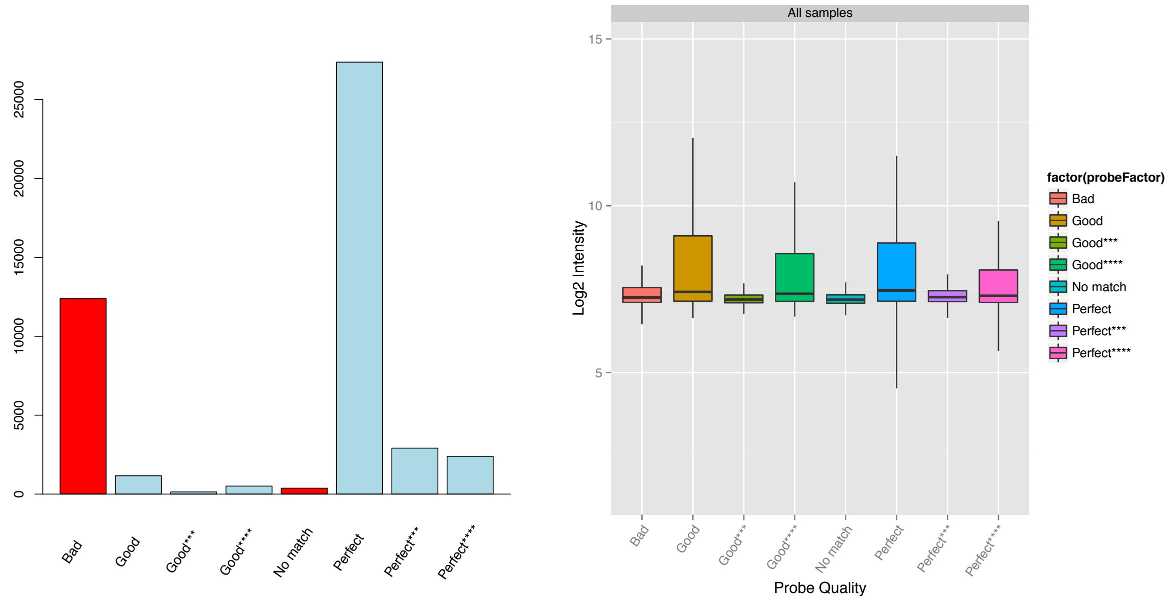


**Supplement Figure 6.** The distribution of probes according to their quality scores. On the left a total of 12755 probes are classified either as 'Bad' or 'No Match' reflecting the frequencies of the two red columns in the histogram on the left. After filtering those probes, 34476 probes are available for downstream analysis. On the right is shown the relationship between probes quality and their intensities.

**Supplement Table 8.** The combinations of transformation and normalization methods tested to select the best one for our dataset.

| **Methods** | **Implementation** |
|---|---|
| log2 + scale normalization | limma |
| log2 + quantile normalization | limma |
| log2 + cyclic loess normalization | limma |
| log2 + quantile normalization | beadarray |
| log2 + cubic splines normalization | beadarray |
| log2 + scale normalization | beadarray |
| log2 + rank invariant normalization | beadarray |
| vsn normalization | beadarray |
| vsn normalization | vsn |
| log2 + quantile normalization | lumi |
| log2 + robust spline normalization | lumi |
| log2 + simple scaling normalization | lumi |
| log2 + loess normalization | lumi |
| log2 + rank invariant normalization | lumi |
| cubic root + quantile normalization | lumi |
| cubic root + robust spline normalization | lumi |
| cubic root + simple scaling normalization | lumi |
| cubic root + loess normalization | lumi |

*Summary*

NASFinder was used to provide a systems-wide yet more detailed analysis of gene expression at 6, 48, 96, 192, and 384 hours after induction of differentiation in pre-adipocytes. Developing methods and tools for systems analysis should provide more comprehensive understanding of the behavior of cellular processes of complex systems over time.

## 4. References

1. Margetic, S., Gazzola, C., Pegg, G.G. & Hill, R.A. Leptin: a review of its peripheral actions and interactions. *Int J Obesity* **26**, 1407-1433 (2002).
2. Friedman, J. Leptin at 20: an overview. *Journal of Endocrinology* **223**, T1-T8 (2014).
3. Bijland, S., Mancini, S.J. & Salt, I.P. Role of AMP-activated protein kinase in adipose tissue metabolism and inflammation. *Clin Sci* **124**, 491-507 (2013).
4. Arad, M. et al. Transgenic mice overexpressing mutant PRKAG2 define the cause of Wolff-Parkinson-White syndrome in glycogen storage cardiomyopathy. *Circulation* **107**, 2850-2856 (2003).
5. Fischer-Posovszky, P., Newell, F.S., Wabitsch, M. & Tornqvist, H.E. Human SGBS cells - a unique tool for studies of human fat cell biology. *Obes Facts* **1**, 184-189 (2008).
6. Varma, V. et al. Metabolic fate of fructose in human adipocytes: a targeted 13C tracer fate association study. *Metabolomics*, 1-16 (2014).
7. Kim, H.S. et al. Oleanolic acid suppresses resistin induction in adipocytes by modulating Tyk-STAT signaling. *Nutrition Research* **33**, 144-153 (2013).
8. Kabir, S.M., Lee, E.S. & Son, D.S. Chemokine network during adipogenesis in 3T3-L1 cells: Differential response between growth and proinflammatory factor in preadipocytes vs. adipocytes. *Adipocyte* **3**, 97-106 (2014).
9. Mariani, O. et al. JUN oncogene amplification and overexpression block adipocytic differentiation in highly aggressive sarcomas. *Cancer Cell* **11**, 361-374 (2007).
10. Knebel, B. et al. A mutation in the c-fos gene associated with congenital generalized lipodystrophy. *Orphanet journal of rare diseases* **8**, 119 (2013).
11. Hulsmans, M. et al. Interleukin-1 receptor-associated kinase-3 is a key inhibitor of inflammation in obesity and metabolic syndrome. *PLoS One* **7**, e30414 (2012).
12. Wang, T., Wang, Y. & Yamashita, H. Evodiamine inhibits adipogenesis via the EGFR-PKCalpha-ERK signaling pathway. *Febs Lett* **583**, 3655-3659 (2009).
13. White, U.A. & Stephens, J.M. Transcriptional factors that promote formation of white adipose tissue. *Mol Cell Endocrinol* **318**, 10-14 (2010).
14. Mutch, D.M., Rouault, C., Keophiphath, M., Lacasa, D. & Clement, K. Using gene expression to predict the secretome of differentiating human preadipocytes. *Int J Obes (Lond)* **33**, 354-363 (2009).
15. von der Heyde, S., Fromm-Dornieden, C., Salinas-Riester, G., Beissbarth, T. & Baumgartner, B.G. Dynamics of mRNA and polysomal abundance in early 3T3-L1 adipogenesis. *BMC Genomics* **15**, 381 (2014).
16. Yang, W.L. et al. Arp2/3 complex regulates adipogenesis by controlling cortical actin remodelling. *Biochemical Journal* **464**, 179-192 (2014).
17. Song, D.D. et al. Protein tyrosine phosphatase 1B inhibits adipocyte differentiation and mediates TNFalpha action in obesity. *Biochim Biophys Acta* **1831**, 1368-1376 (2013).
18. Kim, Y.J., Kim, H.J., Chung, K.Y., Choi, I. & Kim, S.H. Transcriptional activation of PIK3R1 by PPARgamma in adipocytes. *Mol Biol Rep* **41**, 5267-5272 (2014).

19. Tse, M.C.L., Liu, X., Yang, S.R., Ye, K.Q. & Chan, C.B. Fyn Regulates Adipogenesis by Promoting PIKE-A/STAT5a Interaction. *Molecular and Cellular Biology* **33**, 1797-1808 (2013).
20. Yuan, Z. et al. PPARgamma and Wnt Signaling in Adipogenic and Osteogenic Differentiation of Mesenchymal Stem Cells. *Current stem cell research & therapy* (2015).
21. Guo, B. et al. The clock gene, brain and muscle Arnt-like 1, regulates adipogenesis via Wnt signaling pathway. *Faseb J* **26**, 3453-3463 (2012).
22. Varma, V. et al. Metabolic fate of fructose in human adipocytes: a targeted C tracer fate association study. *Metabolomics* **11**, 529-544 (2015).
23. Muller, M.R. & Rao, A. NFAT, immunity and cancer: a transcription factor comes of age. *Nat Rev Immunol* **10**, 645-656 (2010).
24. Feng, T., Szabo, E., Dziak, E. & Opas, M. Cytoskeletal disassembly and cell rounding promotes adipogenesis from ES cells. *Stem Cell Rev* **6**, 74-85 (2010).
25. Herman, M.A., She, P., Peroni, O.D., Lynch, C.J. & Kahn, B.B. Adipose tissue branched chain amino acid (BCAA) metabolism modulates circulating BCAA levels. *J Biol Chem* **285**, 11348-11356 (2010).
26. Kitsy, A. et al. Effects of leucine supplementation and serum withdrawal on branched-chain amino acid pathway gene and protein expression in mouse adipocytes. *PLoS One* **9**, e102615 (2014).
27. McLean, J.R., Chaix, D., Ohi, M.D. & Gould, K.L. State of the APC/C: organization, function, and structure. *Critical reviews in biochemistry and molecular biology* **46**, 118-136 (2011).
28. Hauguel-de Mouzon, S. et al. The GLUT3 glucose transporter isoform is differentially expressed within human placental cell types. *J Clin Endocrinol Metab* **82**, 2689-2694 (1997).
29. Newgard, C.B. Interplay between Lipids and Branched-Chain Amino Acids in Development of Insulin Resistance. *Cell Metab* **15**, 606-614 (2012).
30. Jain, P. et al. Systems Biology Approach Reveals Genome to Phenome Correlation in Type 2 Diabetes. *Plos One* **8** (2013).
31. Hogervorst, F., Kuikman, I., von dem Borne, A.E. & Sonnenberg, A. Cloning and sequence analysis of beta-4 cDNA: an integrin subunit that contains a unique 118 kd cytoplasmic domain. *Embo J* **9**, 765-770 (1990).
32. Liu, J. et al. Changes in integrin expression during adipocyte differentiation. *Cell Metab* **2**, 165-177 (2005).
33. Kim, S.J. et al. Transcriptome analysis and promoter sequence studies on early adipogenesis in 3T3-L1 cells. *Nutrition research and practice* **1**, 19-28 (2007).
34. Mathieu, P.S. & Loboa, E.G. Cytoskeletal and focal adhesion influences on mesenchymal stem cell shape, mechanical properties, and differentiation down osteogenic, adipogenic, and chondrogenic pathways. *Tissue engineering. Part B, Reviews* **18**, 436-444 (2012).
35. Urs, S. et al. Effect of soluble Jagged1-mediated inhibition of Notch signaling on proliferation and differentiation of an adipocyte progenitor cell model. *Adipocyte* **1**, 46-57 (2012).
36. Lai, P.Y., Tsai, C.B. & Tseng, M.J. Active form Notch4 promotes the proliferation and differentiation of 3T3-L1 preadipocytes. *Biochem Biophys Res Commun* **430**, 1132-1139 (2013).
37. Jung, K.M. et al. Regulated intramembrane proteolysis of the p75 neurotrophin receptor modulates its association with the TrkA receptor. *J Biol Chem* **278**, 42161-42169 (2003).
38. Strong, A.L., Gimble, J.M. & Bunnell, B.A. Analysis of the Pro- and Anti-Inflammatory Cytokines Secreted by Adult Stem Cells during Differentiation. *Stem Cells International* **2015**, 12 (2015).
39. Santibanez, J.F. & Kocic, J. Transforming growth factor-beta superfamily, implications in development and differentiation of stem cells. *Biomolecular concepts* **3**, 429-445 (2012).

40. Song, B.Q. et al. Inhibition of Notch Signaling Promotes the Adipogenic Differentiation of Mesenchymal Stem Cells Through Autophagy Activation and PTEN-PI3K/AKT/mTOR Pathway. *Cellular physiology and biochemistry : international journal of experimental cellular physiology, biochemistry, and pharmacology* **36**, 1991-2002 (2015).
41. Elshorbagy, A.K., Kozich, V., Smith, A.D. & Refsum, H. Cysteine and obesity: consistency of the evidence across epidemiologic, animal and cellular studies. *Curr Opin Clin Nutr Metab Care* **15**, 49-57 (2012).
42. Ueki, I. & Stipanuk, M.H. 3T3-L1 Adipocytes and Rat Adipose Tissue Have a High Capacity for Taurine Synthesis by the Cysteine Dioxygenase/Cysteinesulfinate Decarboxylase and Cysteamine Dioxygenase Pathways. *Journal of Nutrition* **139**, 207-214 (2009).
43. Armstrong, A.R., Laws, K.M. & Drummond-Barbosa, D. Adipocyte amino acid sensing controls adult germline stem cell number via the amino acid response pathway and independently of Target of Rapamycin signaling in Drosophila. *Development* **141**, 4479-4488 (2014).
44. Green, C.R. et al. Branched-chain amino acid catabolism fuels adipocyte differentiation and lipogenesis. *Nat Chem Biol* **12**, 15-21 (2016).
45. Pierre, S., Bats, A.S. & Coumoul, X. Understanding SOS (Son of Sevenless). *Biochem Pharmacol* **82**, 1049-1056 (2011).
